# Stable Isotope-Labeled Lipidomics to Unravel the Heterogeneous Development Lipotoxicity

**DOI:** 10.3390/molecules23112862

**Published:** 2018-11-02

**Authors:** Lu-Min Shih, Hsiang-Yu Tang, Ke-Shiuan Lynn, Cheng-Yu Huang, Hung-Yao Ho, Mei-Ling Cheng

**Affiliations:** 1 Healthy Aging Research Center, Chang Gung University, Taoyuan 33302, Taiwan; snoopy68512@yahoo.com.tw (L.-M.S.); tangshyu@mail.cgu.edu.tw (H.-Y.T.); chenyu7015@gmail.com (C.-Y.H.); hoh01@mail.cgu.edu.tw (H.-Y.H.); 2 Department of Biomedical Sciences, College of Medicine, Chang Gung University, Taoyuan 33302, Taiwan; 3 Metabolomics Core Laboratory, Chang Gung University, Taoyuan 33302, Taiwan; 4 Department of Mathematics, Fu-Jen Catholic University, New Taipei City 24205, Taiwan; 128171@mail.fju.edu.tw; 5 Department of Medical Biotechnology and Laboratory Science, College of Medicine, Chang Gung University, Taoyuan 33302, Taiwan; 6 Clinical Metabolomics Core Laboratory, Chang Gung Memorial Hospital, Taoyuan 33305, Taiwan; 7 Graduate Institute of Biomedical Sciences, College of Medicine, Chang Gung University, Taoyuan 33302, Taiwan

**Keywords:** non-alcoholic fatty liver disease, lipotoxicity, fatty acid metabolism, stable isotope tracers, lipidomics, myriocin

## Abstract

Non-alcoholic fatty liver disease (NAFLD) as a global health problem has clinical manifestations ranging from simple non-alcoholic fatty liver (NAFL) to non-alcoholic steatohepatitis (NASH), cirrhosis, and cancer. The role of different types of fatty acids in driving the early progression of NAFL to NASH is not understood. Lipid overload causing lipotoxicity and inflammation has been considered as an essential pathogenic factor. To correlate the lipid profiles with cellular lipotoxicity, we utilized palmitic acid (C16:0)- and especially unprecedented palmitoleic acid (C16:1)-induced lipid overload HepG2 cell models coupled with lipidomic technology involving labeling with stable isotopes. C16:0 induced inflammation and cell death, whereas C16:1 induced significant lipid droplet accumulation. Moreover, inhibition of de novo sphingolipid synthesis by myriocin (Myr) aggravated C16:0 induced lipoapoptosis. Lipid profiles are different in C16:0 and C16:1-treated cells. Stable isotope-labeled lipidomics elucidates the roles of specific fatty acids that affect lipid metabolism and cause lipotoxicity or lipid droplet formation. It indicates that not only saturation or monounsaturation of fatty acids plays a role in hepatic lipotoxicity but also Myr inhibition exasperates lipoapoptosis through ceramide in-direct pathway. Using the techniques presented in this study, we can potentially investigate the mechanism of lipid metabolism and the heterogeneous development of NAFLD.

## 1. Introduction

Cellular lipids are a heterogeneous category of compounds. Based on the diversity of chemical structures and biosynthetic pathways, lipids are mainly divided into eight categories: fatty acyls, glycerolipids, glycerophospholipids, sphingolipids, sterol lipids, prenol lipids, saccharolipids, and polyketides [1]. These ubiquitous molecules perform several pivotal functions, including energy storage, cell membrane modulation, “fat-soluble” vitamin formation, and hormonal regulation as well as act as a cellular massager [2]. Thus, perturbation of lipid metabolism would contribute to obesity development and an increase in the incidences of associated diseases such as cardiovascular disease, hypertriglyceridemia, type 2 diabetes, and non-alcoholic fatty liver disease (NAFLD) [3,4]. 

Non-alcoholic fatty liver disease is characterized by lipid accumulation in the liver without excessive alcohol consumption. Given that hepatic lipotoxicity is implicated in dysfunction of hepatocytes owing to lipid overload, in vitro exposure of liver cells with high concentrations of specific free fatty acids (FFAs) results in lipid overload-aggravated inflammatory responses such as steatohepatitis, non-alcoholic steatohepatitis (NASH), and lipotoxicity [5,6]. Previous studies have substantiated that FFAs contribute to various manifestations of lipotoxicity [5,7], but little is known about the effects of saturation or monounsaturation of FFAs on lipid overload-induced metabolic changes and hepatocyte injury, particularly because most researches until now have been based on molecular phenomena or biomarker findings [8,9,10].

Lipidomics is a branch of metabolomics and focuses on the comprehensive identification and quantification of all lipids in a biological system [11]. Recently, lipidomics has been applied to various diseases such as neurodegenerative diseases, cardiovascular diseases, metabolic syndrome, cancer, and infectious diseases to reveal the role of lipids in cellular functions, various disease pathologies, and disease biomarker discovery [12]. 

The stable isotope-labeling approaches are capable of providing dynamic information on lipid metabolism [13]. However, in most lipidomics analyses, the dynamics of lipid metabolism reflecting alterations in synthesis and degradation of individual lipid species is less investigated.

Moreover, generation of a comprehensive pattern covering as many stable isotope-labeled lipid species as possible is a prerequisite to identify unexpected alterations in lipid metabolism. Recently, several studies have revealed that liquid chromatography–mass spectrometry (LC–MS) technique and stable isotopes were used to investigate alterations in lipid biosynthesis. Various categories of lipids such as phospholipids and triacylglycerols (TGs) could be measured by isotope incorporation after treatment, thereby augmenting our knowledge of lipid metabolism [13,14,15]. 

In this study, palmitic acid induced inflammation and palmitoleic acid induced lipid droplet accumulation. This indicates that saturation or monounsaturation of fatty acids plays a role in hepatic lipotoxicity. To differentiate the complex dynamics of lipid metabolism between the palmitic acid-overload and palmitoleic acid-overload HepG2 cells, LC–MS and stable isotopes were used for investigating lipid content in cells exposed to 0.3 mM ^13^C16-palmitic acid or 0.3 mM ^13^C16-palmitoleic acid for 4, 8, and 16 h. By utilizing stable isotope-labeled tracers to distinguish *de novo* lipogenesis from pre-existing lipid categories, it is possible to comprehend the regulation of lipid metabolism and transport of lipids in the palmitic acid- and palmitoleic acid-treated metabolic perturbation; this technique also supports measurement of the dynamic changes via the lipidomics approach [16] Stable isotope-labeled lipidomics reveals the effects of specific fatty acids on lipid metabolism and their roles in lipotoxicity or lipid droplet formation. This strategy in the present study greatly facilitates the elucidation of lipid metabolism and heterogeneous development of NAFLD. Myriocin (Myr) is an antibiotic isolated from the thermophilic fungus *Myriococcum albomyces* [17]. Blocking the initial step in the sphingolipid biosynthetic pathway by serine palmitoyltransferase inhibitor, Myr could potentially lead to modulate various downstream sphingolipid species [18]. Thus, combining the finding of significant metabolites in FFAs treatment with the assessment of Myr inhibition, it could shed light on the role of ceramide function related to palmitic acid-induced lipoapoptosis.

## 2. Results

### 2.1. The Effect of Saturation in Free Fatty Acids

**Figure 1:**
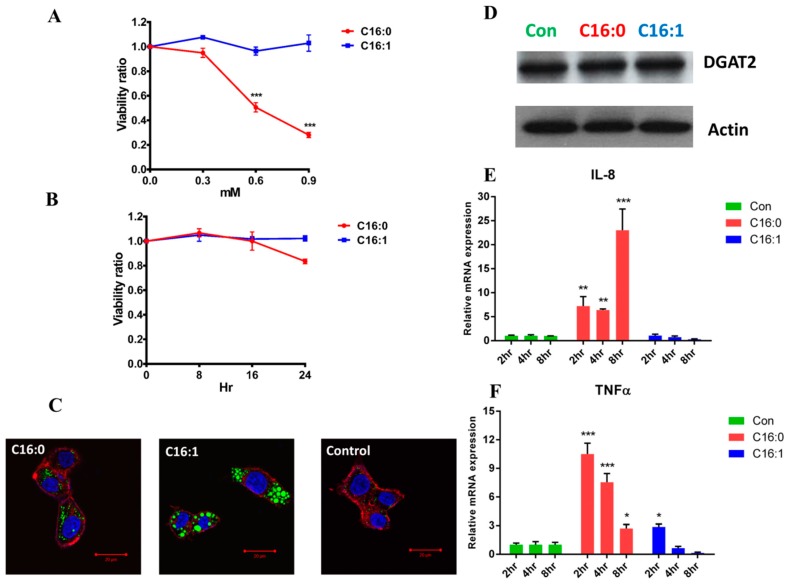
The viability of FFA-treated HepG2 cells was detected using fluorescent cell viability assays. (**A**) The dosage-dependent 24-h incubation with 0.3, 0.6, and 0.9 mM FFAs and (**B**) the time-dependent incubation with 0.3 mM FFAs. These results were representative of at least three separate experiments. (**C**) Observation of lipid droplets in HepG2 cell using BODIPY (493/503) staining. The cells were incubated with 0.3 mM FFAs for a 16-h treatment. Green illumination represented neutral lipid, red illumination represented F-actin, and blue illumination represented the nucleus. (**D**) The protein expression level of diglyceride acyltransferase 2 (DGAT2) was determined by western blot analysis. (**E**) The TNF-α and (**F**) IL-8 mRNA expression level after treatment with 0.3 mM FFAs for 2-, 4-, and 8-h incubation. The value of relative mRNA expression was normalized to the control Actin gene. (* *p* < 0.05, ** *p* < 0.01, *** *p* < 0.001).

Compared with the monounsaturated FFAs (C16:1), saturated FFAs (C16:0) possessed a higher cytotoxicity in the dose-dependent experiment and decreased the HepG2 cell viability to the range from 20% to 50% after a 24-h treatment (Figure 1A). A similar result of cell viability was observed in previous reports [19,20]. In addition, all HepG2 cells incubated with 0.3 mM FFAs in the time-course experiment, except the palmitoleic acid-treated cells (Figure 1B), exhibited over-accumulation of fats and a decrease in cell viability to approximately 80% after the 24-h treatment. This result indicated that the palmitoleic acid did not seem to be toxic to HepG2 cells during the treatment period. Compared with control cells, we observed significant accumulation of intracellular lipid droplets in HepG2 cells after 16-h incubation and staining with BODIPY 493/503. Moreover, increased accumulation of lipid droplets was observed in monounsaturated FFA (C16:1)-treated cells than in saturated FFA (C16:0)-treated ones (Figure 1C). The diglyceride acyltransferase 2 (DGAT2) protein expression levels were not significantly different between palmitoleic acid (C16:1) or palmitic acid (C16:0)-treated cells (Figure 1D). While investigating significant correlation of cell viability with inflammation, we noted that mRNA levels of tumor necrosis factor-α (TNF-α) and Interleukin-8 (IL-8) increased in palmitic acid (C16:0)-treated cells but not in palmitoleic acid (C16:1)-treated ones (Figure 1E,F). Owing to such results, we further used palmitic acid and palmitoleic acid to investigate and elucidate the effects of saturation of FFAs on lipid overload-induced metabolic changes using LC–MS analyses. 

### 2.2. Differential Lipidomics Profiling between Palmitic Acid- and Palmitoleic Acid-Treated HepG2 Cells

**Figure 2:**
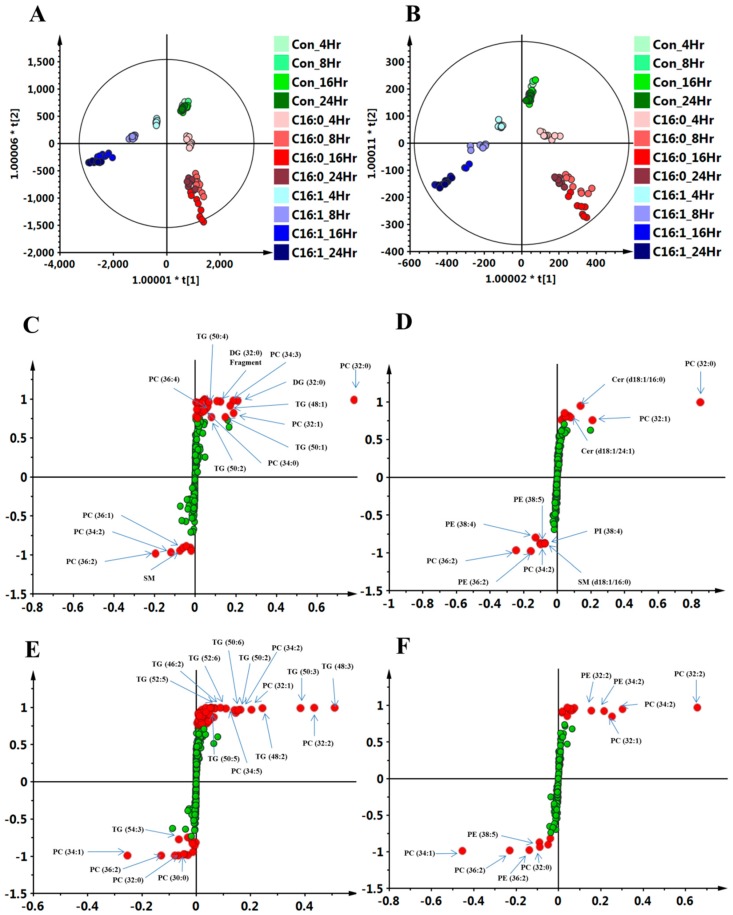
FFAs induced metabolic change in HepG2 cells performed by LC–TOFMS-based lipidomics analysis in ESI-positive and -negative modes. HepG2 cells treated with or without 0.3 mM palmitic acid (C16:0) or palmitoleic acid (C16:1) for 4, 8, 16, and 24 h were subjected to the modified Folch’s method for lipid extraction, followed by chromatography and TOF-MS analysis in ESI-positive and-negative modes. All molecular features of HepG2 cells (*n* = 9 per group, biological triplicates and technical triplicates) were determined by the Progenesis QI software, and subsequently, data processing and statistical analysis were performed by SIMCA-P. The orthogonal partial least squares discriminant analysis (OPLS-DA) score plot was generated from all the hydrophobic metabolite profiles and depicted in panels (**A**) and (**B**), demonstrating positive and negative modes separately. The ellipse shown in the model represents Hotelling’s T2 test with 95% confidence interval. The data of control or C16:0- and C16:1-treated cells (16-h treatment) were re-analyzed using OPLS-DA, and the metabolites with a significant difference between the normal control and C16:0 or between the normal control and C16:1-treated cells were represented in S-plots (**C**) (C16:0, ESI-positive mode), (**D**) (C16:0, ESI-negative mode), (**E**) (C16:1, ESI-positive mode), and (**F**) (16:1, ESI-negative mode). The red points were marked as the significant metabolites in the realm, *p*(corr) > 0.75 and *p*(corr) < −0.75, respectively.

Liver hepatocellular cells, HepG2 cells, were incubated with 0.3 mM palmitic acid and palmitoleic acid for 4, 8, 16, and 24 h, and the organic (lower) layer was removed by the Folch extraction method for LC–MS analysis. After performing calculations using the Progenesis QI software, we obtained 385 and 442 variables in the electrospray ionization (ESI)-positive mode of palmitic acid and palmitoleic acid treatment, respectively. There were 285 and 283 variables in the ESI-negative mode of palmitic acid and palmitoleic acid treatment, respectively. These data were then applied for SIMCA-P analysis, respectively. The orthogonal projections to latent structures discriminant analysis (OPLS-DA) score plot showed remarkable separation among control, palmitic acid, and palmitoleic acid groups in both ESI-positive or -negative lipid profiling (Figure 2A,B). For further detailed study, the control group (untreated group) and 16-h palmitic acid-treated and palmitoleic acid-treated groups in both ESI-positive and ESI-negative lipid profiling were re-analyzed and the OPLS-DA plots (data not shown) and S-plots were represented in Figure 2C (C16:0, ESI-positive mode), Figure 2D (C16:0, ESI-negative mode), Figure 2E (C16:1, ESI-positive mode), and Figure 2F (C16:1, ESI-negative mode). There were only 17 and 11 metabolites with variable importance in the projection (VIP) scores (>1.0), significant differences (*p* < 0.001) and in S-plot (*p*(corr)) selected range (>0.75 and <−0.75) between the 16 h palmitic acid-treated and the control groups in ESI-positive and -negative modes (Tables S1 and S2). This result of untargeted analysis revealed phospholipids, ceramide (Cer), and glycerolipids to be important discriminators of the control and the palmitic acid-treated groups. There were only 26 and 10 metabolites with VIP scores (>1.0), significant differences (*p* < 0.001) and in S-plot (*p* (corr)) selected range (>0.75 and <−0.75) between the 16-h palmitoleic acid-treated and the control groups in ESI-positive and ESI-negative modes (Tables S3 and S4). This result of untargeted analysis revealed phospholipids and glycerolipids to be important discriminators of the controls and the palmitoleic acid-treated groups. The set of significant metabolites were cooperated with and as an indicator or an orientation of stable isotope-labeled experiment.

### 2.3. Lipids Dynamic Changes in ^13^C16-Palmitic Acid- and ^13^C16-Palmitoleic Acid-Treated HepG2 Cells

**Figure 3:**
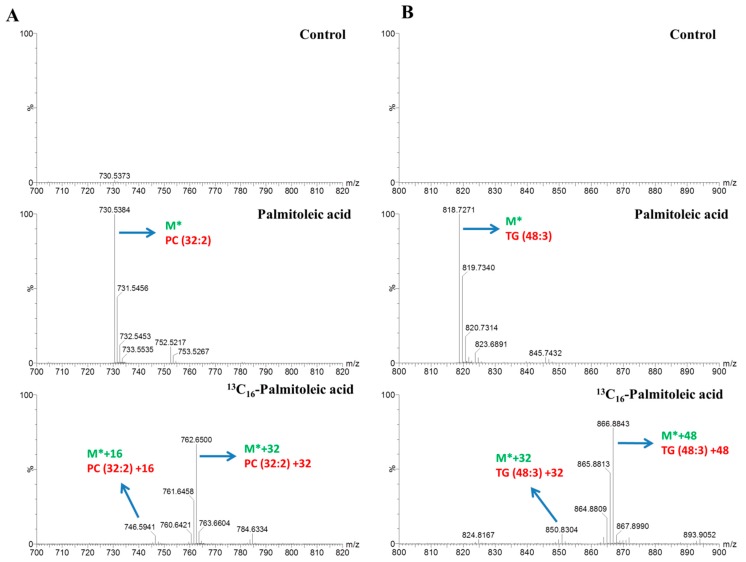
Illustration of the stable isotope-labeled C16:1 metabolite mass spectrum. (**A**) The peaks of PC (32:2) (M*) and its isotopomers (M* + 16 or M* + 32) detected in HepG2 cells incubated with 0.3 mM palmitoleic acid and ^13^C16-labeled palmitoleic acid, respectively, after16-h treatment. (**B**) The peaks of TG (48:3) (M*) and its isotopomers (M* + 16 or M* + 48) detected in HepG2 cells incubated with 0.3 mM palmitoleic acid and 13C16-labeled palmitoleic acid, respectively, after 16-h treatment. M* means the *m*/*z* in positive or negative mode.

**Figure 4:**
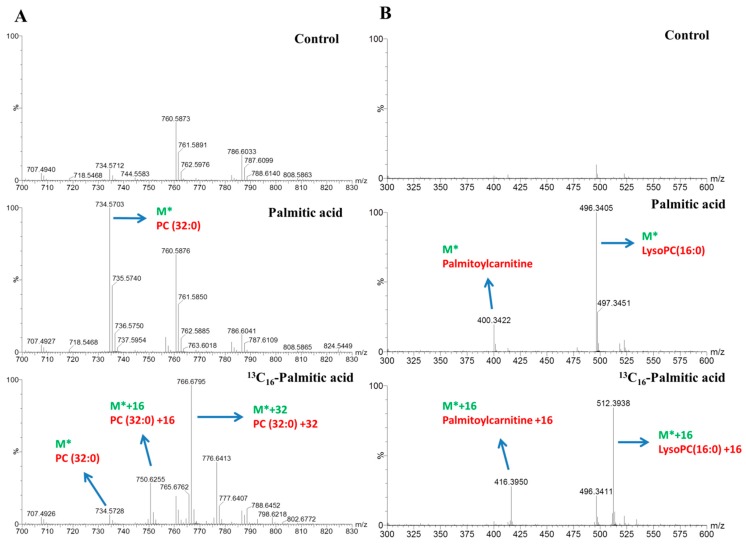
Illustration of the stable isotope-labeled C16:0 metabolite mass spectra. (**A**) The peaks of PC (32:0) (M*) and its isotopomers (M* + 16 or M* + 32) detected in HepG2 cells incubated with 0.3 mM palmitic acid and ^13^C16-labeled palmitic acid respectively after 16-h treatment. (**B**) The peaks of palmitoylcarnitine and LysoPC (16:0) (M*) and its isotopomers (M* + 16) detected in HepG2 cells incubated with 0.3 mM palmitic acid and 13C16-labeled palmitic acid, respectively, after 16-h treatment. M* means the *m*/*z* in positive or negative mode.

The dynamics of lipid metabolism of the palmitic acid- and palmitoleic acid-treated cells was investigated, using LC–MS and stable isotope-labeling, to reflect the alteration in the synthesis and degradation of individual lipid species. After incubating with 0.3 mM stable isotope-labeled tracers for 4-, 8-, and16-h treatments and detection using LC–MS analysis, we identified significant changes in the metabolites and determined each of the ^13^C16-labeled signals using data matrices. For instance, the peak at *m*/*z* 730.5384 corresponded to phosphatidylcholine (PC) (32:2) (M*) and increased the ion intensity over time in the palmitoleic acid-treated group (Figure 3A). The peaks at *m*/*z* 746.5941 (M* + 16), 762.6500 (M* + 32) represented distinct molecules of PC (32:2) containing one, two equivalents ^13^C16-labeled signals of palmitoleic acid. The peak at *m*/*z* 818.7271 corresponded to TG (48:3) (M*) and increased the ion intensity over time in the palmitoleic acid-treated group (Figure 3B). The peaks at *m*/*z* 850.8304 (M* + 32), and 866.8843 (M* + 48) represented distinct molecules of TG (48:3) containing two and three equivalent ^13^C16-labeled signals of palmitoleic acid. The result displayed that most of the TG (48:3) originated from de novo lipogenesis. In the palmitic acid-treatment group, the peaks at *m*/*z* 734.5703 (M*), 750.6255 (M* + 16), and 766.6795 (M* + 32) represented distinct molecules of PC (32:0), containing one and two equivalent ^13^C16-labeled signals of palmitic acid (Figure 4A). The peaks at *m*/*z* 400.3422 (M*) and 416.3950 (M* + 16) or at 496.3405 (M*) and 512.3938 (M* + 16) represented distinct molecules of palmitoylcarnitine (C16:0-carnitine) or LysoPC (16:0), respectively, containing one equivalent ^13^C16-labeled signals of palmitic acid (Figure 4B). The lipids labeled with isotopes according to the strategy used in our study are shown in Table 1 and Table 2.

### 2.4. Mapping of Lipid Metabolism in Palmitic Acid- and Palmitoleic Acid-Treated HepG2 Cells

**Figure 5:**
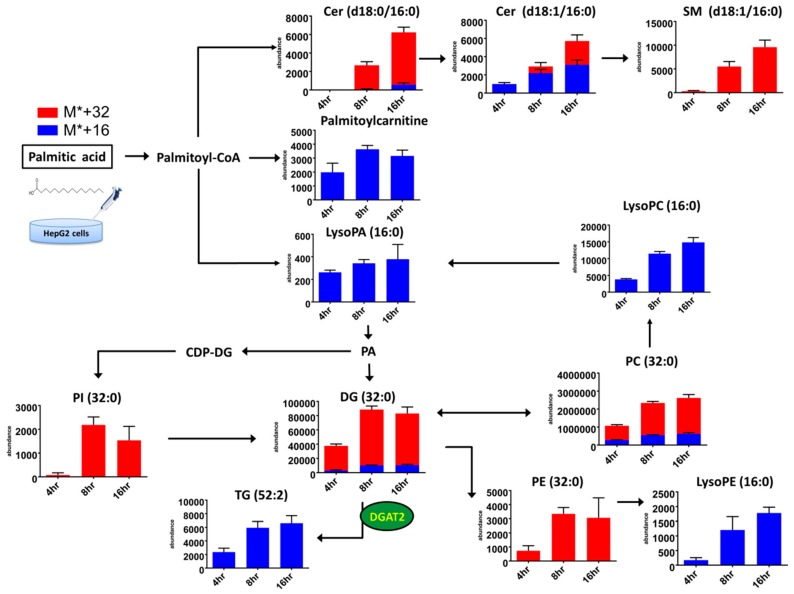
Metabolic pathways of and dynamic changes in lipid classes in HepG2 cells incubated for 4, 8, and 16 h with 0.3 mM ^13^C16-palmitic acid. The cellular abundances of metabolites labeled with the stable isotope were marked as blue (M* + 16), and red (M* + 32) bar charts. (mean ± SD, *n* = 18, triplicates).

**Figure 6:**
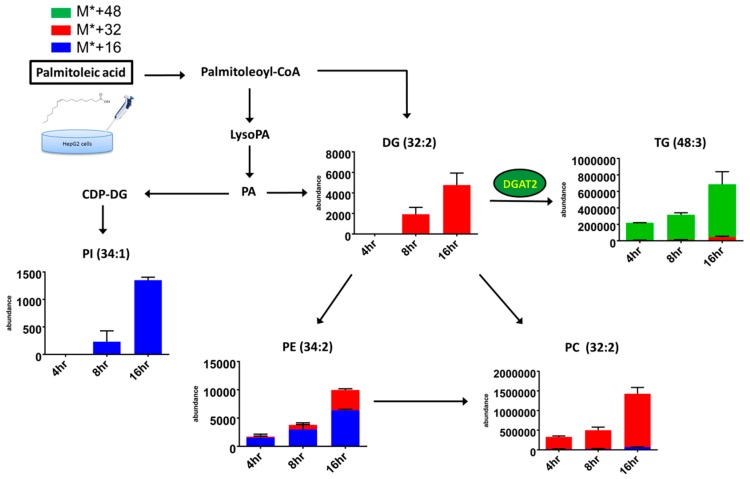
Metabolic pathways of and dynamic changes in lipid classes in HepG2 cells incubated for 4, 8, and 16 h with 0.3 mM ^13^C16-palmitoleic acid. The cellular abundances of metabolites labeled with the stable isotope were marked as blue (M* + 16), red (M* + 32), and green (M* + 48) bar charts. (mean ± SD, *n* = 18, triplicates).

A dynamic picture of lipid metabolism is generated, reflecting a lipid pattern consisting of all the detected lipids ([^12^C + ^13^C] labeled and unlabeled species) and percentage of ^13^C isotopomers within one labeled species (^13^C/[^12^C + ^13^C]) (Table 1 and Table 2). Moreover, the present approach provides comprehensive information on dynamic changes in lipid fingerprints, reflecting the rates of degradation and biosynthesis of lipids. Therefore, we plotted bar charts of the represented lipids and the *x* axis represented the cellular abundance of labeled isotopomers belonging to each of the represented lipid categories and their associated metabolic pathways in palmitic acid treatment (Figure 5) and palmitoleic acid treatment (Figure 6). Significant incorporation of ^13^C16-palmitic acid over time was found for diacylglycerol (DG), TG, phosphatidylinositol (PI), Dihydroceramide (dHCer), Cer, sphingomyelin (SM), PC, phosphatidylethanolamine (PE), lysoPC, lysophosphatidic acid (LysoPA), lysoPE and palmitoylcarnitine. Significant incorporation of ^13^C16-palmitoleic acid over time was observed for TG, DG, PC, PI, and PE. The results demonstrated that robust isotope-labeled TG accumulation was in the palmitoleic acid-treated group, yet major dynamic changes in lipid metabolism were observed for isotope-labeled DG and isotope-labeled Cer in the palmitic acid-treated group. The specific PC (32:2) and PC (32:0) were significantly increased in palmitoleic acid-and palmitic acid-treated groups, respectively.

### 2.5. Aggravating Cell Death through the Inhibition of De Novo Sphingolipid Synthesis

**Figure 7:**
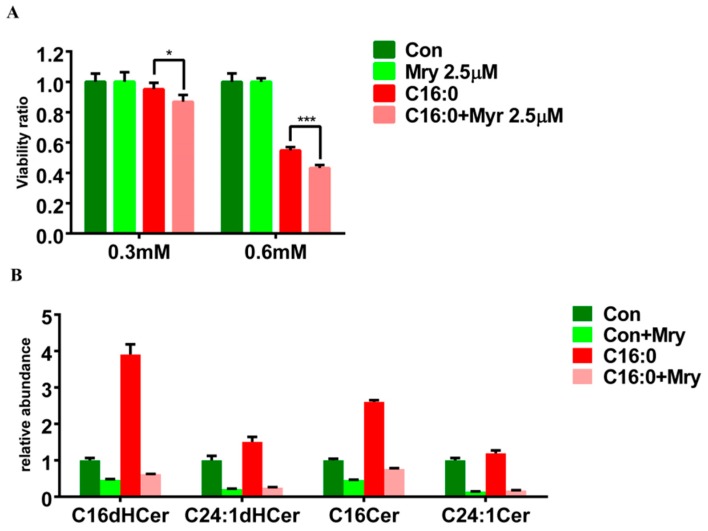
Myriocin (Myr) decreased the cell viability and ceramide levels of palmitic acid-treated HepG2 cells. (**A**) The 0.3 and 0.6 mM palmitic acid 24-h incubation with or without Myr. These results were representative of at least three separate experiments. (* *p* < 0.05, ** *p* < 0.01, *** *p* < 0.001) (**B**) The relative abundance of targeted ceramides in 0.3 mM palmitic acid 24-h incubation with or without Myr. Metabolic data (*n* = 36, triplicates) were detected using TQ-MS in ESI-positive mode and normalized to internal control, C17 ceramide, and total cell protein levels.

According to Figure 5 and Table S2, these results demonstrated that ceramide synthesis was a unique mechanism in palmitic acid treatment. We utilized a very potent inhibitor of serine palmitoyltransferase, Myr to block the de novo sphingolipid synthesis. In Figure 7A, palmitic acid (C16:0)-treated cells obtained decreasing cell viability when cells were cultured with increasing levels of FFA for 24 h. Moreover, palmitic acid (C16:0)-treated cells were followed by addition of Myr. Either in 0.3 mM or in 0.6 mM treated cells, addition of Myr caused much significant cell mortality than only FFA treatment. C16 and C24:1 ceramides in Table S2 were pointed out that both of them were significant metabolites in palmitic acid treated HepG2 cells. So, we detected and relatively quantified these targeted ceramides by using TQ-MS. After normalized to internal control and total protein levels, Figure 7B showed that the levels of targeted ceramide species were higher and induced by FFA treatment; yet, all targeted ceramide levels were decreased by addition of Myr in both control and palmitic acid treatment. Thus, this result indicated that Myr inhibition did not mitigate but deteriorate lipoapoptosis in palmitic acid treated cells through ceramide in-direct pathway.

## 3. Discussion

The pivotal purposes of this study were to understand saturation or monounsaturation of fatty acids on lipid overload-induced metabolic changes in HepG2 cells using stable isotope-labeled tracers. Levels of pro-inflammatory cytokine such as TNF-α and IL-8 are usually higher in NAFLD patients and thus promote apoptosis and recruitment of additional inflammatory cells to the liver, thereby deteriorating hepatic inflammation [21,22,23,24,25]. Therefore, according to cell viability assay and mRNA expressions of inflammatory factors (TNF-α and IL-8), palmitic acid induced maximum apoptosis in HepG2 cells; moreover, analysis of inflammatory marker expression implicated that these cells in vitro might mimic the in vivo conditions of cells in patients with NASH [26,27]. On the contrary, palmitoleic acid has been reported to have numerous health benefits, including increase in cell membrane fluidity and inhibition of oncogenesis as well as reduction in diabetes- and heart disease-associated inflammation and other health adversities [28]. A previous report had also revealed that palmitoleic acid functions as an adipose tissue-derived lipid hormone that stimulates muscle insulin action and suppresses hepatosteatosis [29]. Consistent with the findings of our study, it suggested that a high proportion of palmitic acid but not palmitoleic acid might represent a cellular model of steatosis in which saturated FFAs promote an acute deleterious effect of fat over-accumulation.

BODIPY 493/503 staining revealed significant lipid droplet accumulations in palmitoleic acid-treated HepG2 cells, and detected elevated levels of isotope labeled-triglycerides. The results suggest that triglyceride accumulation protected against unsaturated fatty acid-induced lipotoxicity [6,30]. In the group of cells treated with excess palmitic acid, lipid droplet accumulation was also detected but leading to cell death. Because of poor conversion of DGs to TGs, saturated fatty acid-overload treatment leads to DG accumulation, and Cer synthesis—dysfunction of homeostasis between sphingolipids and glycerophospholipids. These metabolic disturbances contribute to lipotoxic response and also greatly elevate DG, Cer, and PC levels. Additionally, lipotoxicity was observed in HepG2 cells overloaded with saturated FFAs [5,20,26]. In the previous reports, DG and Cer are thought to be signaling lipids and caused cell toxicity when their intercellular concentrations were augmented [31]. Moreover, lipid overload inducing sphingolipid-dependent oxidative stress and mitochondrial dysfunction led to cell death [32,33,34]. However, our results demonstrated that inhibition of ceramide synthesis by Myr resulted in decreasing the levels of targeted ceramide species but increasing cell death. We also detected DG and palmitoylcarnitine levels in palmitic acid and addition of Myr treated cells (data not show). DG was retained in the same highest levels but palmitoylcarnitine was increased in the addition of Myr treated cells. Acylcarnitines accumulation has been observed in type 2 diabetes and obesity, and related to mitochondrial dysfunction and metabolic syndrome [35,36]. Previous report shows that excessive palmitoylcarnitine not only induced pro-inflammatory but also increased cell death in PC3 cells [37]. Blocking of the carnitine palmitoyl transferase-1, a mitochondrial enzyme of acyl carnitines formation, can significant mitigate cardiac apoptosis induced by co-incubation of carnitine and palmitic acid [38]. Especially, increasing concentration of palmitoyl CoA can decrease the granulosa cell viability [39]. Although the lipidomic profiling without the detection of acyl CoA, we could assume that palmitoyl CoA might stay in the highest level as a lipotoxic intermediate to deteriorate the effect of cell death in Myr treated cells. Therefore, to juxtapose Cer and DG, we revealed that accumulation of DG plays the key role in lipoapoptosis than Cer distribution and acylcarnitine pertaining to downstream inflammation or mitochondrial dysfunction could be vital for the aggravating lipoapoptosis.

Moreover, the recent paper reported that acylceramide is metabolized from Cer and sequestrated in lipid droplets through DGAT2 [40] and we observed lipid droplet accumulation in both types of FFA-treated groups. According to these empirical evidences, de novo sphingolipid synthesis might provide one of protecting way for cells to attenuate palmitic acid induced lipoapoptosis through ceramide in-direct pathway.

To shed light on and gain broader insights into the complex dynamics of lipid metabolism in HepG2 cells by investigating the effect of saturation or monounsaturation of FFAs on lipid perturbation and injury of hepatocytes, we utilized stable isotope-labeled FFAs. For instance, some metabolites such as FA-carnitines and Lyso-phospholipids were not used in FFA-overloading treatment until isotope-labeled FFA analyses came into practice. Although we observed lipid droplet accumulation in both types of FFA-treated groups and no significant difference in the protein levels of DGAT2, an essential enzyme of TG synthesis [41,42], the major changes noted with regard to glycerolipid metabolism in both groups were distinctive. This distinction might be due to the difference in the kinetic conversion rate owing to the lipid structure. By combining our approach with the significant candidates from the untargeted lipidomics approach, in the present study, we could elucidate which pivotal mediators participate in NASH mechanism and offer a flawless method for measuring dynamic changes through the lipidomics approach.

Given that metabolomics research has some limitations (such as dynamic flux analysis) [16], we utilized stable isotope-labeled lipidomics to provide deeper insights regarding alterations in the lipid metabolism of specific fatty acid that affect lipid metabolism and cause metabolic perturbations. A recent study [43] involving the use of a stable isotope tracer, ^13^C_5_-glutamine, to quantify cellular fluxomes unveiled the metabolites of glutamine metabolic pathway in fumarate hydratase-deficient cells. In our study, we demonstrated that palmitic acid-treated cells had high conversion rates for palmitoylcarnitine, Cer, phospholipids and DG (Table 1); palmitoleic acid-treated cells possessed high conversion rates for phospholipids, DG, and TG (Table 2). The palmitic acid-treated cells in our study demonstrated that overloading cells with FFA caused a shift in the lipid metabolic pathway to undergo three different pathways to resolve the high concentration of palmitic acid in vitro (Figure 5). On the contrary, palmitoleic acid treatment allowed cells to directly generate glycerolipids in vitro. The pathway followed by stable isotope-labeled lipids also correlates with saturation or monounsaturation of fatty acid, and resulted in lipotoxicity or lipid droplet formation. Thus, stable isotope-labeled tracing demonstrated that robust isotope labeled TG accumulation occurred in the palmitoleic acid-treated group, but major dynamic changes in isotope-labeled DG, FA-carnitine, and Cer during lipid metabolism occurred in the palmitic acid-treated group. Hence, we believe that the present strategy has a promising potential to investigate lipid metabolism and heterogeneous development of NALFD.

## 4. Materials and Methods

### 4.1. Reagents and Chemicals

Dulbecco’s modified Eagle’s medium (DMEM), fetal bovine serum, penicillin–streptomycin antibiotic solution, and trypsin- ethylenediaminetetraacetic acid (EDTA) were obtained from GIBCO (Grand Island, NY, USA). Boron-dipyrromethene (BODIPY) stain (BODIPY 493/503 (D3922), Hoechst 33342 (H1399) and Alexa Fluor 594 phallodin(A12382)) was purchased from Invitrogen Molecular Probes (Eugene, OR, USA). Formaldehyde (37%), and isopropanol were acquired from Sigma-Aldrich (St. Louis, MO, USA). LC–MS grade water, acetonitrile, and methanol were obtained from the Fluka (Muskegon, MI, USA). Chloroform, formic acid, and ammonium formate were purchased from Merck (Billerica, MA, USA). Palmitic acid and palmitoleic acid were obtained from the NU-CHEK (Elysian, MN, USA). The ^13^C16-palmitic acid was purchased from Sigma-Aldrich, and ^13^C16-palmitoleic acid was purchased from Cambridge Isotope Laboratories (Tewksbury, MA, USA). C16, C17, C24:1 ceramide and C16, C24:1 dihydroceramide standards were obtained from Avanti Polar Lipids (Alabaster, AL, USA). Myriocin was purchased from Cayman Chemical (Ann Arbor, MI, USA). All the following experiments are also demonstrated as a workflow chart in Figure S1.

### 4.2. Free Fatty Acid Treatment

The FFA-containing medium was prepared by diluting 150 mM FFA stock solution (dissolved in isopropanol) in DMEM supplemented with 1% fatty acid-free bovine serum albumin (Sigma), and followed by incubating overnight at 37 °C [26].

### 4.3. Cell Viability Assay

HepG2 cell line (ATCC, USA) were cultured in DMEM with low glucose (1 g/L) and 10% fetal bovine serum and 1% PenStrepl (100 unit/mL penicillin plus 100 µg/mL streptomycin sulfate in 0.85% saline) and were incubated under 5% CO_2_ at 37 °C. For sub-culturing, cells were dispensed with Puck’s buffer solution (Invitrogen) and detached by treatment with 0.25% trypsin-EDTA at 37 °C [5,26]. The HepG2 cell viability was measured using the fluorescent cell viability assay. In the dose-response assay, approximately 2 × 10^5^ HepG2 cells were cultured in 12-well plates for 24 h at 37 °C by treatment with different concentrations of FFAs (0, 0.3, 0.6, and 0.9 mM). In addition, 2 × 10^5^ HepG2 cells were treated with 0.3 mM FFAs for 8, 16, and 24 h in the time-course of effect. Moreover, in the assessment of ceramide inhibition, 2 × 10^5^ HepG2 cells were cultured for 24 h with 2.5 µm myriocin (stock solution dissolved in dimethyl sulfoxide) adding into DMEM with 0.3 mM and 0.6 mM palmitic acid. At the end of treatments, cells were fixed with 3.7% formaldehyde solution, and followed by addition of Hoechst 33342 (at 1:250 dilution) for 2 h staining. The illumination of fluorescence was detected using IN Cell Analyzer 1000 (GE Healthcare Bio-Sciences, Chicago, IL, USA).

### 4.4. BODIPY (493/503) Staining

After treatment with 0.3 mM FFAs for 16 h, there were 2 × 10^5^ HepG2 cells stained by BODIPY 493/503 (at 1:20,000 dilution) for observing the distribution of the lipid droplets through the LSM 510 Meta Confocal Microscope (Zeiss, Oberkochen, Germany) [44,45].

### 4.5. Relative mRNA expression

HepG2 cells (10^6^) were treated with 0.3 mM FFAs for 2, 4, and 8 h and subsequently harvested for total RNA isolation. The total RNA was extracted using TRIzol reagent (Life Technologies, Carlsbad, CA, USA), and quantified using NanoDrop (Implen, Munich, Germany). One microgram of RNA was reversely transcribed to cDNA using RevertAid First Stand cDNA Synthesis Kit (K1622, Fermentas, Waltham, MA, USA).The quantitative, real-time RT-PCR (qRT-PCR) was performed with SsoFast EvaGreen Supermix reagent (Bio-Rad, Hercules, CA, USA) using a CFX96 Touch Real-Time PCR Detection System (Bio-Rad). The thermal cycle program was set as follows: 95 °C for 3 min, 39 cycles for amplification at 98 °C for 5 s and 60 °C for 5 s. The following primer pairs were used for qRT-PCR analysis: human interleukin-8 (IL-8; forward: 5′-CTTTCAGAGACAGCAGAG-3′ and reverse: 5′-CTAAGTTCTTTAGCACTCC-3′), human tumor necrosis factor-alpha (TNF-α forward: 5′-CCTGTGAGGAGGACGAAC-3′ and reverse: 5′-CGAAGTGGTGGTCTTGTTG-3′), and the housekeeping Actin gene (forward primer: 5′-GAGATGCGTTGTTACAGGAA-3′ and reverse: 5′-GCATTACATAATTTACACGAAAGC-3′). The relative mRNA expression was normalized to the control actin gene.

### 4.6. Western Blot Analysis

Thirty microgram of whole-cell lysate quantified by using a SpectraMax 340PC384 microplate reader (Molecular Devices, San José, CA, USA) was isolated from 10^6^ HepG2 cells, which were incubated with 0.3 mM FFAs for a 16-h treatment, and was resolved using SDS-PAGE (Bio-Rad). After electrophoresis, proteins were transferred to a poly-vinylidene fluoride membrane (Millipore, Burlington, MA, USA) for 2 h at 4 °C at 300 mA. Western blots were incubated with anti-diglyceride acyltransferase 2 (DGAT2) antibodies (1:2000; Abcam, Cambridge, UK). Immunoblotting was conducted using secondary antibodies (anti-rabbit or anti-mouse) and protein bands were detected using ECL reagent and the ChemiDoc MP Imaging System (Bio-Rad).

### 4.7. Sample Preparation for Lipidomics

In brief, total lipids were extracted from 10^6^ HepG2 cells (control, palmitic acid, palmitoleic acid, ^13^C16-palmitic acid, ^13^C16-palmitoleic acid and myriocin treatment) using the modified Folch method [46,47]. The lipid extract containing internal control (C17 ceramide) was dissolved in isopropanol/acetonitrile/water (2:1:1, *v*/*v*/*v*) mixture. After vortexing (30 s, 4 times) and centrifuging (12,000 rpm, for 20 min at 4 °C) the mixture, the supernatant was transferred into a vial for LC–MS analysis. Samples per group were biological triplicates (*n* = total number of biological replicates) and each of biological triplicates was detected three times for technical triplicates (triplicates).

### 4.8. Profiling and Identification of Lipid Species Using Liquid Chromatography System Coupled with Time-Of-Flight Mass Spectrometry

Mass spectrometry analysis was performed using the ultra-performance liquid chromatography (UPLC) system coupled with time-of-flight mass spectrometry (TOF-MS; Waters, Milford, MA, USA). Chromatographic separation was performed on an ACQUITY UPLC CSH C18 column (2.1 mm × 100 mm × 1.7 µm). Column temperature was maintained at 55 °C. For metabolite profiling, the mobile phase A was acetonitrile/water (60:40, *v*/*v*) and the mobile phase B was isopropanol/acetonitrile (90:10, *v*/*v*), and both solvents contained 10 mM ammonium formate and 0.1% formic acid. The flow rate was 0.4 mL/min, and the solvent gradient was as follows: 0–2 min, 40–43% solvent B; 2–2.1 min, 43–50% solvent B; 2.1–12 min, 50–54% solvent B; 12–12.1 min, 54–70% solvent B; 12.1–18 min, 70–99% solvent B; 18–18.1 min, 99–40% solvent B; 18.1–20 min, 40% solvent B [48].

Mass spectrometric analysis was performed using the Waters Synapt High Definition Mass Spectrometry (HDMS) system operating in positive- and negative-ion ESI mode. The capillary voltage was set at 2700 V in ESI-positive mode and 2000 V in ESI-negative mode and cone voltage was set at 35 V, respectively. Desolvation gas flow rate was set at 800 L/h, and cone gas flow was maintained at 25 L/h. The desolvation and source temperatures were set at 400 °C and 100 °C, respectively. MS data were collected in centroid mode over a range of 20–990 *m*/*z* at a rate of 0.1 scan/s. Leucine-enkephalin was used as the reference compound. LockSpray frequency was set at 0.5 s and was averaged over 10 scans for correction.

Data matrices were determined utilizing the Progenesis QI software V.2.3 (Waters, Milford, MA, USA) by the extracted *m*/*z* value, retention time (RT), and ion intensity. OPLS-DA and S-plot analysis were also performed using the Pareto scaling method and SIMCA-P software. Significance of variables was selected under the conditions of *p*(corr) value of >0.75 or <−0.75, a *p* value of <0.001, and VIP values of >1.0.

Metabolite identification was performed by searching the extracted data against the METLIN (http://metlin.scripps.edu/index.php), LIPID MAPS (http://www.lipidmaps.org/), Human Metabolome Database (HMDB) (http://www.hmdb.ca/), and in-house database (Figure S6).

For achieving an isotope labeling pattern, as shown in Figure 3 and Figure 4 pertaining to the unique information using PC (32:0), PC (32:2), TG (48:3), palmitoylcarnitine and LysoPC (16:0) as examples, all adducts of metabolites (M + H^+^, M + NH_4_^+^, M + Na^+^, M − H^−^, M − H + FA^−^) were marked as M* in isotope-labeled FAs sections and the peak lists were evaluated and picked the ion doublets (M*, M* + 16), triplets (M*, M* + 16, M* + 32), or quadruplets (M*, M* + 16, M* + 32, M* + 48) in MS spectra defined as potential isotopomers using our in-house Matlab program with *m*/*z* tolerance of 30 ppm (^13^C − ^12^C = 1.003355 was used) and RT shift tolerance of 0.1 min. All filtered isotopomers belonging to one lipid species were then matched against the METLIN. Additionally, isotope labeling in conjunction with MS/MS also allows the determination of the labeling position. The significantly altered metabolites were represented as bar charts based on their relative ion intensity and their roles in lipid metabolism were mapped.

### 4.9. Relative Quantification of Targeted Ceramide Species Using Liquid Chromatography System Coupled with Electrospray Ionization Tandem Mass Spectrometry

Mass spectrometry analysis was performed using the UPLC system coupled with tandem mass spectrometry (TQ-MS; Waters). Chromatographic separation was performed on an ACQUITY UPLC BEH C18 column (2.1 mm × 100 mm × 1.7 µm). Column temperature was maintained at 60 °C. For optimized parameters, the mobile phase A was acetonitrile/water (40:60, *v*/*v*) and the mobile phase B was isopropanol/acetonitrile (90:10, *v*/*v*), and both solvents contained 10 mM ammonium formate. The flow rate was 0.45 mL/min, and the solvent gradient was as follows: 0–10 min, 40–99% solvent B; 10–10.1 min, 99–40% solvent B; 10.1–12 min, 40% solvent B.

Mass spectrometric analysis was performed using the Waters Xevo TQ-S system operating in positive-ion ESI mode. The capillary voltage was set at 1500 V and cone voltage was set at 30 V. Desolvation gas flow rate was set at 900 L/h, and cone gas flow was maintained at 150 L/h. The desolvation and source temperatures were set at 550 °C and 120 °C, respectively. MS data were collected in centroid mode at a rate of 0.1 scan/s. C16, C24:1 dihydroceramide and C16, C24:1 ceramide standards were dissolved in isopropanol/acetonitrile/water (2:1:1, *v*/*v*/*v*) mixture and major MS/MS fragment patterns were determined [49].

Data matrices were determined utilizing the MassLynx software V.4.1 (Waters, Milford, MA, USA) by the extracted *m*/*z* value, retention time (RT), and ion intensity. Targeted ceramide data were normalized to internal control (C17 ceramide) and total cell protein levels.

## Figures and Tables

**Table 1:** Metabolites were isotope labeled in ^13^C16-palmitic acid-treated (^13^C16:0) HepG2 cells.

POS		4 h	8 h	16 h
		%	%	%
**Palmitoylcarnitine**	M*	-	-	-
	M* + 16	99.5	99.3	99.5
**LysoPC (16:0)**	M*	-	-	-
	M* + 16	61.1	78.4	84.0
**PC (32:0)**	M*	-	-	-
	M* + 16	24.1	23.1	23.5
	M* + 32	69.8	74.2	74.3
**Cer (d18:0/16:0)**	M*	-	-	-
	M* + 16	-	2.5	9.1
	M* + 32	-	97.1	90.7
**Cer (d18:1/16:0)**	M*	-	-	-
	M* + 16	98.0	74.8	53.2
	M* + 32	-	24.4	44.3
**SM (d18:1/16:0)**	M*	-	-	-
	M* + 32	0.2	2.7	5.5
**DG (32:0)**	M*	-	-	-
	M* + 16	8.1	11.6	12.7
	M* + 32	91.8	88.4	87.3
**TG (52:2)**	M*	-	-	-
	M* + 16	3.1	8.6	15.3
**NEG**		%	%	%
**LysoPA (16:0)#**	M*	-	-	-
	M* + 16	92.1	92.1	92.4
**LysoPE (16:0)**	M*	-	-	-
	M* + 16	61.3	81.7	84.8
**PI (32:0)**	M*	-	-	-
	M* + 32	88.3	99.5	99.4
**PE (32:0)**	M*	-	-	-
	M* + 32	98.7	99.7	99.7

HepG2 cells were treated with ^13^C16-palmitic acid for 4, 8, and 16 h. Metabolites were detected using LC–TOFMS in ESI-positive (POS) or -negative (NEG) modes. The metabolites were evaluated and picked to be ion doublets (M*, M* + 16), or triplets (M*, M* + 16, M* + 32) and defined as potential isotopomers. The labeled fractions (^13^C/[^12^C + ^13^C], %) of these represented lipids are shown. (# is marked as targeted metabolite with *m*/*z* that detected in an aqueous extraction of modified Folch’s method and the aqueous phase analysis by LC-MS is described in the Supplementary Materials).

**Table 2:** Stable isotope-labeled metabolites in ^13^C16-palmitoleic acid-treated (^13^C16:1) HepG2 cells.

POS		4 h	8 h	16 h
		%	%	%
**PC (32:2)**	M*	-	-	-
	M* + 16	6.7	5.8	4.9
	M* + 32	93.1	94.2	95.1
**DG (32:2)**	M*	-	-	-
	M* + 16	-	-	-
	M* + 32	-	99	99.6
**TG (48:3)**	M*	-	-	-
	M* + 32	3.8	4.3	6.4
	M* + 48	96.2	95.7	93.6
**PI (34:1)**	M*	-	-	-
	M* + 16	-	95.9	99.3
**PE (34:2)**	M*	-	-	-
	M* + 16	58.6	75.8	63
	M* + 32	7.2	20	35.3

HepG2 cells were treated with ^13^C16-palmitoleic acid for 4, 8, and 16 h. Metabolites were detected using LC–TOFMS in ESI-positive (POS) or -negative (NEG) modes. The metabolites were evaluated and picked to be ion doublets (M*, M* + 16), triplets (M*, M* + 16, M* + 32), or quadruplets (M*, M* + 16, M* + 32, M* + 48) and defined as potential isotopomers. The labeled fractions (^13^C/[^12^C + ^13^C], %) of these represented lipids are shown.

## Supplementary Materials

The following are available online, Table S1: Significant changes in metabolites produced by palmitic acid-treated (FA 16:0) HepG2 cells (in ESI-positive mode), Table S2: Significant changes in metabolites produced by palmitic acid-treated (FA16:0) HepG2 cells (in ESI-negative mode), Table S3: Significant changes in metabolites produced by palmitoleic acid-treated (FA 16:1) HepG2 cells (in ESI-positive mode), Table S4: Significant changes in metabolites produced by palmitoleic acid-treated (FA 16:1) HepG2 cells (in ESI-negative mode), Table S5: Stable isotope-labeled metabolites in 13C16-palmitic acid (isoFA 16:0)- and 13C16-palmitoleic acid (isoFA 16:1)-treated HepG2 cells, Table S6: In-house database, Supplementary Figure, Figure S1: The workflow of FFAs treated HepG2 cell experiments, Supplementary materials and methods, Sample preparation for aqueous phase, Aqueous phase analyzed by LC-MS.
