# Supplementary material for: Stable Isotope-Labeled Lipidomics to Unravel the Heterogeneous Development Lipotoxicity
Source: Molecules. 2018 Nov 2;23(11):2862. doi: 10.3390/molecules23112862 (PMC6278256; doi:10.3390/molecules23112862)
Supplement: Supplementary file 1 [file molecules-23-02862-s001.pdf]

molecules

MDPI

Supplementary Materials

Stable isotope-labeled lipidomics to unravel the heterogeneous development lipotoxicity

Firstname Lastname [1], Firstname Lastname [2] and Firstname Lastname [2,*] Lu-Min Shih [1,2,3], Hsiang-Yu Tang [1,2,3], Ke-Shiuan Lynn [6], Cheng-Yu Huang [1,2,3], Hung-Yao Ho [5,7], and Mei-Ling Cheng [1,2,3,4,5,*]

1 Healthy Aging Research Center, Chang Gung University, Taoyuan, 33302, Taiwan
2 Department of Biomedical Sciences, College of Medicine, Chang Gung University, Taoyuan, 33302, Taiwan
3 Metabolomics Core Laboratory, Chang Gung University, Taoyuan, 33302, Taiwan
4 Graduate Institute of Biomedical Sciences, College of Medicine, Chang Gung University, Taoyuan, 33302, Taiwan
5 Clinical Metabolomics Core Laboratory, Chang Gung Memorial Hospital, Taoyuan, 33305, Taiwan
6 Department of Mathematics, Fu-Jen Catholic University, New Taipei City, 24205, Taiwan
7 Department of Medical Biotechnology and Laboratory Science, College of Medicine, Chang Gung University, Taoyuan, 33302, Taiwan
* Correspondence: chengm@mail.cgu.edu.tw; Tel.: +886-3-2118800 (ext.3811); Fax: 886-3-2118700

Supplementary Materials

Supplementary Tables

Table S1. Significant changes in metabolites produced by palmitic acid-treated (FA 16:0) HepG2 cells (in ESI-positive mode)

ID	m/z	CON_4HR	CON_8HR	CON_16HR	CON_24HR	FA16:0_4HR	FA16:0_8HR	FA16:0_16HR	FA16:0_24HR	VIP 16:0 (16HR)	S-Plot 16:0 (16HR)	FA 16:0 P-value (16HR)
PC(32:0)	734.5834	24691.7±1153.3	27339.0±1408.9	28110.2±2361.7	22855.4±694.3	364963.0±56529.1	781911.3±90400.4	935030.0±132598.1	686010.6±50028.2	16.81	-0.99	3.31E-08
DG(32:0)	591.5063	3.5±10.6	0.0±0.0	13.2±30.5	0.0±0.0	12607.8±1720.6	38102.9±5615.1	66384.8±12540.1	50815.8±6556.5	4.52	-0.98	2.48E-07
PC(36:2)	786.6069	69746.6±4876.9	73574.0±3341.9	75936.7±4271.8	75220.0±4247.1	50343.8±3712.0	39227.5±3117.0	18670.2±3815.4	8804.8±1134.6	4.25	0.98	1.72E-15
PC(32:1)	732.5675	79483.9±3415.6	89803.3±3453.2	104516.1±8222.2	110212.9±7997.9	91532.7±7892.1	134392.3±8126.8	169217.6±33023.7	172518.1±14801.4	4.10	-0.82	0.0003
PC(34:3)	756.5590	0.0±0.0	0.0±0.0	10.2±30.6	5.3±16.0	12197.3±3268.7	41992.1±7255.3	52537.9±11588.1	30804.8±2764.3	4.00	-0.97	8.22E-07
TG(48:1)	822.7662	9264.1±835.0	9650.5±1758.9	10796.9±1862.2	10367.5±1652.7	27619.3±4552.5	40287.3±9300.8	58718.1±16568.7	78618.0±38348.0	3.72	-0.91	2.17E-05
TG(50:1)	850.8030	29585.0±11971.7	32632.6±4718.9	25574.7±14775.2	27504.9±12631.2	70014.0±18945.2	88392.5±24498.8	68192.0±22530.2	62405.4±49855.4	3.22	-0.77	0.0002
DG(32:0)Fragment	313.2760	12.9±10.9	15.5±12.6	28.5±21.6	19.6±21.2	4199.4±552.4	12523.2±1045.2	23270.9±5806.1	16238.7±1885.6	2.65	-0.96	2.13E-06
PC(34:2)	758.5779	20933.8±3166.1	21873.1±1557.1	27586.5±1728.5	36324.9±5152.7	12828.2±2012.8	10753.8±920.6	5770.5±2836.6	5519.1±1104.5	2.61	0.97	1.21E-12
Unknown	551.5065	343.1±68.1	365.4±67.3	321.5±43.7	292.3±83.8	3779.6±471.5	11177.4±1655.4	18243.2±4160.7	13581.7±1472.7	2.33	-0.97	1.22E-06
TG(50:2)	848.7869	19857.6±713.4	21855.0±4038.1	28224.3±3044.5	27046.5±3991.7	32164.7±2887.3	36541.8±3099.0	41103.1±7239.7	56213.3±9468.4	1.78	-0.76	0.0005
SM	703.5786	14187.6±958.3	15595.0±1686.5	16674.7±1212.0	13995.0±5313.1	12807.0±1785.2	11664.8±834.7	7707.7±1476.2	6741.6±624.6	1.66	0.95	1.96E-10
TG(50:4)	827.7192	0.0±0.0	39.3±87.5	94.5±172.8	51.7±102.7	2955.2±1095.4	5128.3±891.0	7248.5±1758.0	8709.6±4435.5	1.47	-0.96	1.66E-06
PC(36:1)	788.6203	5272.3±1095.3	7404.4±1194.3	8882.5±1569.5	5315.5±983.2	3283.7±1059.4	4987.3±1226.0	2930.5±1128.1	1241.0±458.6	1.32	0.91	8.18E-08
PC(36:4)	782.5732	2538.4±1431.4	1950.8±537.5	1152.1±665.5	636.0±212.9	5986.0±1800.2	9207.8±942.1	7186.9±2216.7	4821.2±596.4	1.31	-0.90	2.02E-05
PC(34:0)	762.6043	23.5±46.9	57.6±62.1	32.2±66.9	0.0±0.0	365.5±218.2	3972.2±1138.3	4964.1±2259.5	1398.9±815.7	1.16	-0.88	0.0002
Unknown	607.4617	36.2±46.4	69.4±78.7	36.3±59.5	89.6±82.2	1560.9±326.1	2622.1±287.0	3670.1±516.8	3266.9±618.2	1.06	-0.99	2.02E-08

Samples were analyzed using LC–TOFMS in the ESI-positive mode and demonstrated by peak area. Metabolites were selected when VIP values were >1.0, p value was <0.001, and S-plot [p(corr)] value was >0.75 and <−0.75, based on the results of control cells versus 16-hr FA (16:0)-treated cells.

Table S2. Significant changes in metabolites produced by palmitic acid-treated (FA16:0) HepG2 cells (in ESI-negative mode)

ID	m/z	Con_4HR	Con_8HR	Con_16HR	Con_24HR	FA16:0_4HR	FA16:0_8HR	FA16:0_16HR	FA16:0_24HR	VIP 16:0 (16Hr)	S-Plot 16:0 (16Hr)	FA 16:0 P-value (16Hr)
PC(32:0)	778.5676	817.2±212.4	771.0±328.1	950.3±274.4	781.8±300.5	28964.7±5837.3	64428.0±9529.7	69997.9±9882.7	50269.1±3140.9	12.84	-0.99	2.77E-08
PC(36:2)	830.5960	4743.9±739.0	5883.5±773.4	6184.3±1208.3	5792.8±364.6	3150.5±769.2	2187.9±597.1	413.5±248.3	48.1±42.5	3.67	0.97	2.92E-07
PC(32:1)	776.5490	5200.8±1431.9	6672.3±690.6	8367.7±1513.5	8655.0±973.2	7484.5±1866.1	12066.8±2163.5	14002.7±3149.8	13805.8±1215.1	3.25	-0.75	0.0002
PE(36:2)	742.5421	1128.1±370.8	1814.6±524.2	2401.3±427.8	1874.4±550.5	1018.8±444.4	716.5±381.1	37.0±32.6	44.4±50.0	2.36	0.98	1.60E-07
Cer(d18:1/16:0)	582.5118	0.0±0.0	0.0±0.0	3.7±11.0	0.0±0.0	75.9±78.1	816.8±244.7	1952.1±547.9	1586.6±574.4	2.11	-0.95	5.20E-06
PE(38:4)	766.5427	3028.4±975.6	3396.6±601.9	3211.0±918.7	2070.2±507.3	2211.6±644.1	2937.6±809.2	1266.0±698.9	617.6±292.2	1.93	0.80	0.0001
PE(38:5)	764.5281	989.3±410.9	1323.0±289.3	1349.5±394.0	785.8±265.6	747.2±211.2	915.8±484.4	229.9±238.6	42.0±61.9	1.55	0.87	1.80E-06
PC(34:2)	802.5648	566.9±255.8	619.6±370.3	1072.6±404.4	1618.0±431.1	332.0±329.1	135.4±154.4	34.6±53.8	15.9±32.0	1.50	0.89	5.06E-05
Cer(d18:1/24:1)	692.6219	99.2±99.6	81.2±77.5	157.2±99.1	170.8±142.1	63.5±91.0	340.3±200.1	893.5±452.8	666.8±277.1	1.17	-0.79	0.0009
SM(d18:1/16:0)	747.5687	632.2±178.5	805.5±255.3	713.3±244.3	762.9±370.5	599.1±252.4	625.7±241.8	106.4±80.0	125.9±142.2	1.13	0.88	3.95E-05
PI(38:4)	885.5551	812.0±257.1	865.3±305.3	644.1±239.6	321.6±310.0	667.7±278.7	653.8±276.0	55.3±92.1	186.4±143.7	1.11	0.88	3.68E-05

Samples were analyzed using LC–TOFMS in the ESI-negative mode and demonstrated by peak area. Metabolites were selected when VIP values were >1.0, p value was <0.001, and S-plot [p(corr)] value was >0.75) and <−0.75, based on the results of control cells versus 16-hr FA (16:0)-treated cells.

Table S3. Significant changes in metabolites produced by palmitoleic acid-treated (FA 16:1) HepG2 cells (in ESI-positive mode)

ID	m/z	CON_4HR	CON_8HR	CON_16HR	CON_24HR	FA16:1_4HR	FA16:1_8HR	FA16:1_16HR	FA16:1_24HR	VIP 16:1 (16HR)	S-Plot 16:1 (16HR)	FA 16:1 P-value (16HR)
TG(48:3)	818.7439	0.0±0.0	0.0±0.0	78.7±236.0	11.8±35.5	367162.6±46153.9	716009.0±199528.9	1077280.1±114992.2	1232628.4±138803.0	10.97	-0.99	2.78E-09
PC(32:2)	730.5557	9.3±27.9	28.9±43.5	187.8±111.6	843.7±405.9	263883.5±16622.2	484101.9±24051.0	786079.0±80829.7	830999.9±65305.6	9.38	-0.99	2.07E-09
TG(50:3)	846.7766	0.0±0.0	13.7±41.1	5205.8±10646.1	2728.3±7955.0	181411.1±16649.5	375873.9±106497.2	621077.2±83427.3	760821.2±50163.5	8.27	-0.99	1.28E-08
PC(34:1)	760.6002	278665.7±14007.0	312040.0±18403.3	331564.7±26612.7	291665.3±20267.4	189759.5±16927.4	116532.5±9527.5	65276.4±8468.4	52262.8±5325.4	5.46	0.99	1.28E-10
TG(48:2)	820.7564	64.0±115.4	202.5±322.3	2601.4±3917.8	1503.3±3025.3	86035.1±12676.5	164619.8±58477.5	251458.2±31969.7	291774.9±24156.2	5.26	-0.99	8.54E-09
PC(32:1)	732.5675	79483.9±3415.6	89803.3±3453.2	104516.1±8222.2	110212.9±7997.9	181771.7±16502.1	211236.7±15886.5	279813.5±37710.0	307584.7±32882.5	4.36	-0.97	3.39E-07

PC(34:2)	758.5779	20933.8±3166.1	21873.1±1557.1	27586.5±1728.5	36324.9±5152.7	70211.0±5318.3	89081.6±6660.8	141285.8±26257.3	169560.6±15879.7	3.50	-0.97	1.10E-06
TG(50:2)	848.7869	19857.6±713.4	21855.0±4038.1	28224.3±3044.5	27046.5±3991.7	65965.7±8821.1	105294.7±32443.4	129290.1±23259.7	152140.5±9983.8	3.30	-0.96	8.96E-07
Unknown	663.4700	799568.6±50590.3	748757.8±41446.1	712553.4±12742.9	724388.5±39594.9	781494.9±17919.9	787933.0±25870.7	806877.0±27431.1	776469.6±45240.7	3.13	-0.92	1.17E-06
TG(50:6)	823.7055	0.0±0.0	0.0±0.0	0.0±0.0	0.0±0.0	44266.2±1848.7	54724.4±19343.8	86378.1±18990.2	106811.0±14235.9	3.06	-0.96	8.01E-07
PC(36:2)	786.6069	69746.6±4876.9	73574.0±3341.9	75936.7±4271.8	75220.0±4247.1	48508.2±3437.8	25630.9±2778.6	6687.1±3483.4	5350.5±1447.1	2.79	0.99	4.67E-17
PC(34:5)	752.5308	0.0±0.0	0.0±0.0	0.0±0.0	0.0±0.0	10487.6±1185.4	25048.7±1745.3	51101.6±7717.4	60074.8±6155.3	2.38	-0.98	4.30E-08
TG(52:6)	851.7229	0.0±0.0	0.0±0.0	0.0±0.0	0.0±0.0	21177.6±1480.6	25055.5±9595.1	33283.6±2549.0	31723.8±3582.4	1.93	-0.99	1.98E-10
PC(32:0)	734.5834	24691.7±1153.3	27339.0±1408.9	28110.2±2361.7	22855.4±694.3	6387.0±864.7	4392.1±1050.3	2345.0±996.9	2713.5±789.3	1.70	0.99	9.59E-12
Unknown	547.4798	3.6±10.7	0.1±0.3	3.1±9.4	0.0±0.1	4917.3±855.8	11952.8±4200.1	19665.0±2820.3	24361.5±4434.6	1.48	-0.99	2.87E-08
TG(46:2)	792.7162	0.0±0.0	0.0±0.0	0.0±0.0	6.9±20.7	3707.0±710.7	9357.8±3724.4	18936.4±3447.0	24989.2±3494.9	1.44	-0.98	1.85E-07
TG(54:3)	902.8331	25915.6±9851.4	30433.3±5880.7	37430.3±12674.6	39931.7±11616.3	28905.3±2619.0	23884.9±4941.7	14630.0±1127.9	12311.5±1107.7	1.44	0.77	0.0006
Unknown	550.6327	125116.8±9407.2	118161.6±8962.9	110114.6±5635.4	116011.9±5920.7	124734.0±4189.7	125828.7±6165.7	130605.2±6787.1	122276.3±7393.7	1.42	-0.87	3.17E-06
PC(30:0)	706.5420	8858.9±487.9	12793.9±694.9	17418.2±1328.8	14600.5±4574.1	1049.5±463.8	107.1±98.2	29.1±58.3	0.0±0.0	1.40	0.99	1.84E-10
Unknown	845.7478	0.0±0.0	0.0±0.0	0.0±0.0	0.0±0.0	5857.5±698.9	9020.4±3490.4	12943.5±1123.5	13517.7±779.9	1.21	-1.00	5.37E-10
Unknown	839.6623	0.0±0.0	0.0±0.0	0.0±0.0	0.0±0.0	8614.8±651.1	9876.5±3743.9	12443.1±790.1	11916.2±648.6	1.18	-0.99	4.45E-11
Unknown	685.4397	115411.3±8263.3	107016.9±6173.2	102738.3±3241.3	105055.5±7083.1	113082.3±2514.1	112498.1±4602.1	116834.0±5707.9	112247.4±7656.1	1.16	-0.85	8.14E-06
TG(52:5)	853.7338	682.4±656.1	1270.9±608.0	1988.6±1359.4	2680.7±1121.6	8963.2±1606.1	10749.3±3693.3	12987.6±989.1	13420.5±1567.9	1.10	-0.99	1.28E-12
TG(50:5)	825.7069	0.0±0.0	0.0±0.0	17.3±51.9	0.0±0.0	9068.2±1002.6	10869.0±4265.5	11548.7±4572.5	12542.3±1593.2	1.07	-0.90	6.50E-05
Unknown	707.4953	112909.2±7529.7	105143.7±5190.7	100492.3±3647.6	102071.8±5580.9	109586.4±3986.4	112255.4±4089.8	112835.0±5315.6	108636.8±7810.9	1.07	-0.83	3.02E-05
Unknown	522.6005	88313.0±5014.0	82282.2±5019.8	78640.1±1698.9	80146.2±5061.0	86482.6±2150.7	87747.9±3126.4	89225.3±3325.2	85906.9±6027.6	1.04	-0.90	2.49E-07

Samples were analyzed by LC–TOFMS in the ESI-positive mode and demonstrated by peak area. Metabolites were selected when the VIP values were >1.0), p value was <0.001, and S-plot [p(corr)] was >0.75 and <−0.75, based on the results of control cells versus 16-hr FA (16:1)-treated cells.

Table S4. Significant changes in metabolites produced by palmitoleic acid-treated (FA 16:1) HepG2 cells (in ESI-negative mode)

ID		Con_4HR	Con_8HR	Con_16HR	Con_24HR	FA16:1_4HR	FA16:1_8HR	FA16:1_16HR	FA16:1_24HR	VIP 16:1 (16Hr)	S-Plot 16:1 (16Hr)	FA 16:1 P-value (16Hr)
PC(32:2)	774.5402	0.0±0.0	0.0±0.0	0.0±0.0	0.0±0.0	16945.1±1691.8	30765.9±5410.2	51421.7±10287.2	55065.7±3237.7	9.92	-0.97	3.86E-07
PC(34:1)	804.5809	24676.9±2774.5	26436.3±1595.5	27414.5±3075.6	25221.5±1956.2	15470.6±1286.9	9371.6±1673.5	3400.0±1386.7	1901.8±261.1	6.84	0.99	2.22E-10
PC(34:2)	802.5648	566.9±255.8	619.6±370.3	1072.6±404.4	1618.0±431.1	5155.7±594.0	7654.5±1840.2	12206.9±2930.7	13908.3±938.4	4.56	-0.95	2.52E-06
PC(32:1)	776.5490	5200.8±1431.9	6672.3±690.6	8367.7±1513.5	8655.0±973.2	12440.2±1252.7	14161.2±2511.2	17272.9±3876.7	18536.5±1323.5	3.87	-0.85	6.44E-05
PC(36:2)	830.5960	4743.9±739.0	5883.5±773.4	6184.3±1208.3	5792.8±364.6	2061.2±374.7	656.6±312.3	22.3±26.9	14.2±21.4	3.44	0.98	3.28E-07
PE(34:2)	714.5107	5.8±17.4	0.0±0.0	102.4±101.2	42.0±55.0	428.0±318.5	1652.7±779.0	5882.2±1943.2	7378.6±613.7	3.23	-0.92	1.93E-05
PE(32:2)	686.4791	0.0±0.0	0.0±0.0	0.0±0.0	0.0±0.0	122.4±79.4	969.3±277.6	3006.4±977.6	3405.1±333.4	2.34	-0.93	1.54E-05
PE(36:2)	742.5421	1128.1±370.8	1814.6±524.2	2401.3±427.8	1874.4±550.5	748.2±139.0	301.5±148.5	149.4±168.3	56.0±53.6	2.07	0.98	2.67E-08
PE(38:5)	764.5281	989.3±410.9	1323.0±289.3	1349.5±394.0	785.8±265.6	1135.3±321.4	558.9±198.5	271.4±219.0	280.2±132.6	1.37	0.87	2.21E-06
PC(32:0)	778.5676	817.2±212.4	771.0±328.1	950.3±274.4	781.8±300.5	36.8±51.9	3.2±9.6	12.2±24.2	9.4±18.6	1.32	0.93	6.45E-06

Samples were analyzed by LC–TOFMS in the ESI-negative mode and demonstrated by peak area. Metabolites were selected when the VIP values were >1.0, p value was <0.001, and S-plot [p(corr)] was >0.75 and <–0.75, based on the results of control cells versus 16-hr FA (16:1)-treated cells.

Table S5. Stable isotope-labeled metabolites in 13C16-palmitic acid (isoFA 16:0)- and 13C16-palmitoleic acid (isoFA 16:1)-treated HepG2 cells

ID	Adduct	m/z	Con_4hr	IsoFA_4hr	Con_8hr	IsoFA_8hr	Con_16hr	IsoFA_16hr	Source_Mode
Cer(d18:0/16:0)	M+H	540.5368	10.0±0.0	10.0±0.0	10.0±0.0	10.0±0.0	10.0±0.0	12.0±6.1	C16:0_POS
Cer(d18:0/16:0)+16		556.5900	10.0±0.0	10.0±0.0	10.0±0.0	67.2±41.5	10.0±0.0	570.5±201.4	C16:0_POS
Cer(d18:0/16:0)+32		572.6449	10.0±0.0	10.0±0.0	10.0±0.0	2602.0±388.1	10.0±0.0	5665.3±562.5	C16:0_POS
Cer(d18:1/16:0)	M+Na	560.5035	13.8±11.4	10.0±0.0	22.6±25.2	22.4±28.5	14.2±12.5	148.2±188.1	C16:0_POS
Cer(d18:1/16:0)+16		576.5558	10.0±0.0	983.7±176.1	10.0±0.0	2206.3±400.4	10.0±0.0	3119.0±513.7	C16:0_POS
Cer(d18:1/16:0)+32		592.6093	10.0±0.0	10.0±0.0	10.0±0.0	719.0±432.8	10.0±0.0	2595.1±678.2	C16:0_POS
DG(32:0)	M+Na	591.5009	32.8±37.2	22.7±19.5	40.7±47.2	50.0±48.4	22.5±20.9	23.3±39.9	C16:0_POS

DG(32:0)+16		607.5494	10.0±0.0	3066.0±946.7	10.0±0.0	10250.4±561.1	34.4±40.0	10520.0±1232.1	C16:0_POS
DG(32:0)+32		623.6092	22.0±36.1	34571.9±2646.5	10.0±0.0	78315.2±4868.8	75.9±152.7	72488.9±9143.8	C16:0_POS
Palmitoylcarnitine	M+H	400.3426	21.4±12.8	10.7±2.0	138.2±82.0	24.5±17.9	21.9±20.0	16.8±20.5	C16:0_POS
Palmitoylcarnitine+16		416.3970	10.0±0.0	1983.7±654.2	10.0±0.0	3636.0±274.7	10.0±0.0	3161.3±412.6	C16:0_POS
LysoPC(16:0)	M+H	496.3425	559.9±163.1	2425.1±218.6	938.5±108.6	3169.1±297.4	1041.6±220.7	2824.5±333.6	C16:0_POS
LysoPC(16:0)+16		512.3983	12.6±7.9	3807.3±231.4	10.0±0.0	11477.0±645.4	10.0±0.0	14848.5±1430.2	C16:0_POS
PC(30:0)	M+H	706.5519	74787.4±13074.2	35500.5±2830.7	97698.6±11396.6	31924.2±1317.9	117557.3±11476.7	25488.0±2423.8	C16:0_POS
PC(30:0)+16		722.6000	10.0±0.0	16888.1±921.9	10.0±0.0	47835.3±2075.4	10.0±0.0	76511.2±2610.0	C16:0_POS
PC(32:0)	M+H	734.5852	163331.5±27744.0	70026.1±5853.4	180209.6±24506.5	64799.9±3500.6	184835.0±22658.4	60313.7±8229.6	C16:0_POS
PC(32:0)+16		750.6413	4410.9±6627.2	275207.3±26907.2	10.0±0.0	555069.0±25538.4	150.7±266.3	628466.7±63314.6	C16:0_POS
PC(32:0)+32		766.6970	16367.0±24575.1	797537.6±58026.6	381.8±511.5	1782316.9±88497.7	2009.1±2955.7	1990163.6±190498.4	C16:0_POS
PC(34:0)	M+H	762.6056	15951.0±3124.8	10256.9±1257.0	19516.1±2782.9	13648.0±1010.7	18213.6±2748.4	9914.3±1091.7	C16:0_POS
PC(34:0)+16		778.6593	10.0±0.0	8224.1±731.9	10.0±0.0	29634.3±1646.4	10.0±0.0	36073.8±4215.6	C16:0_POS
PC(34:1)	M+H	760.6054	1169330.2±75071.1	866152.1±61126.8	1149825.8±99006.5	667168.0±31426.0	1289932.6±71374.6	523559.5±22612.3	C16:0_POS
PC(34:1)+16		776.6590	10.0±0.0	293350.6±22659.4	10.0±0.0	768481.8±24480.9	10.0±0.0	1153439.7±40080.8	C16:0_POS
PC(34:2)	M+H	758.5771	67936.8±6033.5	45225.4±2348.7	68093.3±9479.2	30949.7±1760.3	74997.2±5847.6	26406.4±1723.0	C16:0_POS
PC(34:2)+16		774.6299	10.0±0.0	19950.3±1317.2	10.0±0.0	39363.5±1277.7	10.0±0.0	57265.7±3493.8	C16:0_POS
PC(34:3)	M+H	756.5633	17986.3±3761.8	312.8±318.6	19999.6±2288.4	80.6±85.8	20271.6±1899.6	77.5±50.5	C16:0_POS
PC(34:3)+16		772.6163	119.4±203.6	8803.2±1510.8	14.8±14.4	9399.8±1687.7	41.0±53.3	8543.1±1751.8	C16:0_POS
PC(34:3)+32		788.6731	2137.9±3222.4	71088.4±4899.4	10.0±0.0	110785.7±8034.0	10.0±0.0	100343.4±7429.9	C16:0_POS
PC(34:4)	M+H	754.5436	50325.9±3864.7	27032.3±1281.1	53672.6±4669.6	12915.7±1380.0	65063.3±2984.2	6213.3±515.8	C16:0_POS
PC(34:4)+16		770.5928	172.5±204.5	14256.7±1144.6	323.9±280.7	25803.6±1550.8	292.8±150.5	31748.3±915.0	C16:0_POS
PC(34:4)+32		786.6461	10.0±0.0	10548.5±1171.7	10.0±0.0	18196.3±713.2	10.0±0.0	26526.2±2064.5	C16:0_POS
PC(36:3)	M+H	784.5896	20258.5±1438.3	14502.6±1180.3	18012.7±2570.3	9609.4±1123.2	18360.1±1485.5	8709.4±342.2	C16:0_POS
PC(36:3)+16		800.6433	10.0±0.0	5455.6±486.8	10.0±0.0	11864.4±518.8	10.0±0.0	17214.3±692.9	C16:0_POS
PC(36:4)	M+H	782.5753	17575.3±959.3	3876.4±501.9	19827.5±2454.3	2066.9±805.2	18058.6±1159.8	2014.1±740.5	C16:0_POS

PC(36:4)+16		798.6296	10.0±0.0	13627.1±1192.9	10.0±0.0	21727.7±1263.0	10.0±0.0	29142.2±1455.9	C16:0_POS
PC(38:6)	M+H	806.5744	6066.5±871.1	516.7±421.1	6678.8±1504.5	114.9±133.7	5895.7±660.0	96.8±133.8	C16:0_POS
PC(38:6)+16		822.6280	10.0±0.0	3651.1±629.7	10.0±0.0	7049.6±358.6	10.0±0.0	9114.9±490.4	C16:0_POS
SM(d18:1/16:0)	M+H	703.5923	220582.8±30495.9	195576.0±12880.1	253114.8±28417.6	201590.3±16795.6	279210.8±23394.4	165626.8±14622.4	C16:0_POS
SM(d18:1/16:0)+32		735.6562	10.0±0.0	303.2±140.2	10.0±0.0	5508.7±1052.9	10.0±0.0	9578.7±1503.3	C16:0_POS
TG(50:1)	M+NH4	850.7936	15662.6±1121.2	11080.9±853.2	17412.0±2467.0	9635.8±665.7	15498.8±1175.0	4401.1±1060.6	C16:0_POS
TG(50:1)+16		866.8460	10.0±0.0	169.8±264.7	10.0±0.0	1247.0±615.7	10.0±0.0	667.5±552.9	C16:0_POS
TG(50:2)	M+NH4	848.7792	22422.6±1363.4	22496.7±1459.3	25650.7±2251.4	19675.8±733.5	22872.7±3050.0	12900.9±755.6	C16:0_POS
TG(50:2)+16		864.8303	10.0±0.0	754.0±381.1	10.0±0.0	1890.5±608.0	10.0±0.0	2410.6±738.8	C16:0_POS
TG(52:2)	M+NH4	876.8211	87856.8±8686.5	72794.5±4502.0	93177.2±9616.4	63522.7±4714.9	80807.6±13766.4	36621.1±2541.0	C16:0_POS
TG(52:2)+16		892.8635	10.0±0.0	2362.3±575.3	10.0±0.0	5949.8±912.7	10.0±0.0	6619.9±1093.2	C16:0_POS
LysoPE(16:0)	M-H	452.2783	10.0±0.0	108.5±102.2	15.0±15.1	269.9±99.3	10.0±0.0	318.8±170.1	C16:0_NEG
LysoPE(16:0)+16		468.3328	10.0±0.0	172.0±85.7	10.0±0.0	1202.8±452.5	10.0±0.0	1777.1±202.0	C16:0_NEG
LysoPA(16:0)	M-H	409.2330	10.0±0.0	22.46±2.22	10.0±0.0	29.58±2.45	10.0±0.0	31.17±9.65	C16:0_NEG
LysoPA(16:0)+16		425.2876	10.0±0.0	262.95±18.59	10.0±0.0	342.94±32.70	10.0±0.0	378.62±131.12	C16:0_NEG
PC(32:1)	M+FA-H	776.5524	56275.0±5746.6	41267.2±3979.5	61714.9±3496.1	33376.0±3080.2	71078.6±6763.5	25298.8±1916.9	C16:0_NEG
PC(32:1)+16		792.6031	10.0±0.0	17486.2±1417.8	10.0±0.0	37603.0±3518.0	10.0±0.0	55051.3±4080.7	C16:0_NEG
PC(32:1)+32		808.6571	10.0±0.0	12313.9±1440.0	10.0±0.0	23141.6±2632.1	10.0±0.0	40263.6±4825.4	C16:0_NEG
PC(36:3)	M+FA-H	828.5793	2851.1±330.2	1363.5±370.9	2376.7±635.4	483.9±202.1	2285.7±424.2	342.6±137.9	C16:0_NEG
PC(36:3)+16		844.6332	10.0±0.0	384.2±204.7	10.0±0.0	1844.4±435.0	10.0±0.0	3534.1±629.7	C16:0_NEG
PE(32:0)	M-H	690.5093	10.0±0.0	10.0±0.0	10.0±0.0	10.0±0.0	10.0±0.0	10.0±0.0	C16:0_NEG
PE(32:0)+32		722.6169	10.0±0.0	731.0±360.0	10.0±0.0	3338.6±454.9	10.0±0.0	3059.1±1421.0	C16:0_NEG
PE(34:0)	M-H	718.5415	80.3±73.2	10.0±0.0	55.1±30.7	10.0±0.0	130.7±77.0	10.0±0.0	C16:0_NEG
PE(34:0)+32		750.6495	10.0±0.0	1505.3±302.7	10.0±0.0	5733.5±730.4	10.0±0.0	6936.0±837.5	C16:0_NEG
PE(34:1)	M-H	716.5256	15749.6±1264.2	12387.9±1008.8	18937.8±1223.1	14811.6±1441.1	19612.8±1185.9	11543.3±409.7	C16:0_NEG
PE(34:1)+16		732.5796	10.0±0.0	4036.9±525.4	10.0±0.0	11859.4±1006.6	10.0±0.0	17463.5±1101.1	C16:0_NEG

PE(36:4)	M-H	738.5101	6663.0±690.5	6419.1±798.5	7138.2±570.9	6288.8±707.4	7466.5±820.6	4552.2±467.6	C16:0_NEG
PE(36:4)+16		754.5640	10.0±0.0	2624.2±482.1	10.0±0.0	6504.8±1072.1	10.0±0.0	9003.6±559.6	C16:0_NEG
PI(32:0)	M-H	809.5215	10.0±0.0	10.0±0.0	10.0±0.0	10.0±0.0	10.0±0.0	10.0±0.0	C16:0_NEG
PI(32:0)+32		841.6296	10.0±0.0	75.6±92.4	10.0±0.0	2182.8±335.9	10.0±0.0	1535.0±588.7	C16:0_NEG
PI(34:1)	M-H	835.5378	646.7±380.7	212.9±111.1	1695.0±372.9	974.2±371.8	2726.9±454.5	823.1±706.0	C16:0_NEG
PI(34:1)+16		851.5912	10.0±0.0	1762.1±471.2	10.0±0.0	7823.9±1283.2	10.0±0.0	7861.0±3280.9	C16:0_NEG
PI(36:4)	M-H	857.5224	336.6±169.6	190.4±89.0	647.0±225.5	77.7±81.0	702.2±221.4	58.9±81.0	C16:0_NEG
PI(36:4)+16		873.5765	10.0±0.0	397.9±209.1	10.0±0.0	2687.9±634.4	10.0±0.0	2054.2±1123.0	C16:0_NEG
DG(32:2)	M+Na	587.4669	10.0±0.0	10.0±0.0	10.0±0.0	10.0±0.0	10.0±0.0	10.0±0.0	C16:1_POS
DG(32:2)+16		603.4402	10.0±0.0	10.0±0.0	10.0±0.0	10.0±0.0	10.0±0.0	10.0±0.0	C16:1_POS
DG(32:2)+32		619.5742	10.0±0.0	10.0±0.0	10.0±0.0	1914.7±671.2	10.0±0.0	4762.6±1163.7	C16:1_POS
PC(32:1)	M+H	732.5704	261362.6±20069.0	137800.2±20248.3	267691.0±6580.3	63903.1±12781.2	399882.2±19686.4	49645.2±5791.7	C16:1_POS
PC(32:1)+16		748.6245	10.0±0.0	198972.1±21074.0	10.0±0.0	250079.6±42882.3	10.0±0.0	706240.8±93155.4	C16:1_POS
PC(32:2)	M+H	730.5546	4132.9±955.2	348.0±150.3	4238.2±23.6	10.0±0.0	10564.1±655.2	10.0±0.0	C16:1_POS
PC(32:2)+16		746.5989	10.0±0.0	22240.5±3012.6	10.0±0.0	29161.0±5412.1	10.0±0.0	69359.9±8237.4	C16:1_POS
PC(32:2)+32		762.6665	10.0±0.0	307009.6±24299.2	10.0±0.0	470442.1±78570.2	10.0±0.0	1357526.2±160707.8	C16:1_POS
PC(34:1)	M+H	760.6043	694019.7±36635.0	457586.7±53195.6	712287.9±8487.7	230274.5±52238.0	954359.2±74985.4	169310.5±22954.7	C16:1_POS
PC(34:1)+16		776.6444	10.0±0.0	3624.4±1169.6	10.0±0.0	6377.5±1933.0	10.0±0.0	26144.6±3498.1	C16:1_POS
PC(34:4)	M+H	754.5421	6072.8±1163.6	941.4±866.0	6567.6±230.9	90.2±113.5	13498.4±1828.6	546.2±204.4	C16:1_POS
PC(34:4)+16		770.5947	10.0±0.0	5557.7±1793.0	10.0±0.0	8151.4±2175.4	10.0±0.0	31120.9±4601.3	C16:1_POS
PC(34:5)	M+H	752.5324	10.0±0.0	10.0±0.0	10.0±0.0	10.0±0.0	16.5±9.2	10.0±0.0	C16:1_POS
PC(34:5)+32		784.6444	10.0±0.0	13616.9±3265.2	10.0±0.0	27261.1±6651.6	10.0±0.0	97049.1±13202.9	C16:1_POS
TG(48:3)	M+NH4	818.7466	10.0±0.0	10.0±0.0	10.0±0.0	10.0±0.0	10.0±0.0	10.0±0.0	C16:1_POS
TG(48:3)+32		850.8450	10.0±0.0	8247.6±245.2	10.0±0.0	13449.3±1296.0	10.0±0.0	44074.3±11518.0	C16:1_POS
TG(48:3)+48		866.9099	10.0±0.0	209471.5±2955.9	10.0±0.0	302325.2±24208.9	10.0±0.0	642343.0±153250.4	C16:1_POS
TG(48:2)	M+NH4	820.7564	6304.0±442.6	5628.8±977.9	6966.2±264.7	2714.7±213.5	8008.6±1445.2	2603.6±1280.4	C16:1_POS

TG(48:2)+32		852.8639	10.0±0.0	40790.6±1755.3	10.0±0.0	62950.3±3779.4	10.0±0.0	147369.7±36567.1	C16:1_POS
TG(50:2)	M+NH4	848.7876	34175.6±3890.3	24212.4±2662.3	37990.5±892.4	14237.3±1022.2	54913.4±7560.3	16490.6±4412.7	C16:1_POS
TG(50:2)+32		880.8791	146.7±122.5	4775.6±473.9	151.2±47.9	5135.8±804.8	327.2±354.6	13901.9±4095.9	C16:1_POS
TG(52:3)	M+NH4	874.7981	18727.3±2060.6	10092.5±751.2	20131.3±334.8	3458.6±742.9	27896.2±4341.3	2223.5±1316.0	C16:1_POS
TG(52:3)+16		890.8469	10.0±0.0	2325.9±257.3	10.0±0.0	2764.2±544.4	10.0±0.0	7901.2±3237.8	C16:1_POS
PC(34:2)	M+FA-H	802.5661	12485.7±2060.9	3848.3±649.8	11458.3±1150.9	861.1±341.5	17435.3±2776.4	138.0±61.3	C16:1_NEG
PC(34:2)+16		818.6198	10.0±0.0	7413.4±1276.2	10.0±0.0	10463.1±565.9	10.0±0.0	13776.8±1018.3	C16:1_NEG
PC(34:2)+32		834.6735	10.0±0.0	1821.7±309.0	10.0±0.0	4812.2±392.0	10.0±0.0	12632.8±1285.1	C16:1_NEG
PE(34:1)	M-H	716.5273	4345.6±791.8	2208.6±918.2	4181.5±834.7	2256.3±605.3	7225.9±1459.7	2181.5±501.6	C16:1_NEG
PE(34:1)+16		732.5809	10.0±0.0	130.5±44.5	10.0±0.0	513.9±329.5	10.0±0.0	3232.9±431.3	C16:1_NEG
PE(34:2)	M-H	714.5121	2494.0±566.3	892.6±122.4	2679.9±264.7	163.1±166.0	4400.5±1177.0	173.4±74.2	C16:1_NEG
PE(34:2)+16		730.5658	10.0±0.0	1529.3±632.8	10.0±0.0	2998.6±855.1	10.0±0.0	6384.1±169.7	C16:1_NEG
PE(34:2)+32		746.6192	10.0±0.0	188.9±88.8	10.0±0.0	792.1±368.3	10.0±0.0	3581.4±217.9	C16:1_NEG
PI(34:1)	M-H	835.5392	10.0±0.0	10.0±0.0	10.0±0.0	10.0±0.0	37.3±47.3	10.0±0.0	C16:1_NEG
PI(34:1)+16	M-H	851.5922	10.0±0.0	10.0±0.0	10.0±0.0	232.3±196.1	10.0±0.0	1351.0±53.3	C16:1_NEG

Samples were analyzed by LC–TOFMS in the ESI-positive or negative mode and demonstrated by peak area.

Table S6. In-house database.

Neutral mass	Compound Id	Description	Formula
268.2402	Fatty acid	10E-heptadecenoic acid	C17H32O2
254.2246	Fatty acid	9Z-hexadecenoic acid	C16H30O2
282.2559	Fatty acid	9Z-octadecenoic acid	C18H34O2
282.2559	Fatty acid	9E-octadecenoic acid	C18H34O2
310.2872	Fatty acid	11Z-eicosenoic acid	C20H38O2
338.3185	Fatty acid	13Z-docosenoic acid	C22H42O2
366.3498	Fatty acid	15Z-tetracosenoic acid	C24H46O2

256.2402	Fatty acid	hexadecanoic acid	C16H32O2
270.2559	Fatty acid	heptadecanoic acid	C17H34O2
284.2715	Fatty acid	octadecanoic acid	C18H36O2
312.3028	Fatty acid	Eicosanoic acid	C20H40O2
340.3341	Fatty acid	docosanoic acid	C22H44O2
368.3654	Fatty acid	tetracosanoic acid	C24H48O2
396.3967	Fatty acid	hexacosanoic acid	C26H52O2
278.2246	Fatty acid	9Z,12Z,15Z-octadecatrienoic acid	C18H30O2
302.2246	Fatty acid	5Z,8Z,11Z,14Z,17Z-eicosapentaenoic acid	C20H30O2
280.2402	Fatty acid	9Z,12Z-octadecadienoic acid	C18H32O2
304.2402	Fatty acid	5Z,8Z,11Z,14Z-eicosatetraenoic acid	C20H32O2
312.2896	Fatty acid	5Z,8Z,11Z,14Z-eicosatetraenoic acid (5,6,8,9,11,12,14,15-d8)	C20H24D8O2
328.2402	Fatty acid	4Z,7Z,10Z,13Z,16Z,19Z-docosahexaenoic acid	C22H32O2
330.2559	Fatty acid	4,7,10,13,16-docosapentaenoic acid	C22H34O2
330.2559	Fatty acid	7,10,13,16,19-docosapentaenoic acid	C22H34O2
306.2559	Fatty acid	8Z,11Z,14Z-eicosatrienoic acid	C20H34O2
306.2559	Fatty acid	5Z,8Z,11Z-eicosatrienoic acid	C20H34O2
332.2715	Fatty acid	7Z,10Z,13Z,16Z-docosatetraenoic acid	C22H36O2
635.4526	Phosphatidylethanolamine	PE 28:0	$C_{33}H_{66}NO_8P$
663.4839	Phosphatidylethanolamine	PE 30:0	$C_{35}H_{70}N_1O_8P$
699.5203	Phosphatidylethanolamine	PEp 34:2	C39H74NO7P
701.5359	Phosphatidylethanolamine	PEp 34:1	C39H76NO7P
703.5516	Phosphatidylethanolamine	PEp 34:0	C39H78NO7P
715.5152	Phosphatidylethanolamine	PE 34:2	C39H74NO8P
717.5309	Phosphatidylethanolamine	PE 34:1	C39H76NO8P
719.5465	Phosphatidylethanolamine	PE 34:0	C39H78NO8P
721.5046	Phosphatidylethanolamine	PEp 36:5	C41H72NO7P
723.5203	Phosphatidylethanolamine	PEp 36:4	C41H74NO7P

727.5516	Phosphatidylethanolamine	PEp 36:2	C41H78NO7P
729.5672	Phosphatidylethanolamine	PEp 36:1	C41H80NO7P
737.4996	Phosphatidylethanolamine	PE 36:5	C41H72NO8P
737.4996	Phosphatidylethanolamine	PE 36:5	C41H72NO8P
739.5152	Phosphatidylethanolamine	PE 36:4	C41H74NO8P
741.5309	Phosphatidylethanolamine	PE 36:3	C41H76NO8P
743.5465	Phosphatidylethanolamine	PE 36:2	C41H78NO8P
745.5622	Phosphatidylethanolamine	PE 36:1	C41H80NO8P
747.5203	Phosphatidylethanolamine	PEp 38:6	C43H74NO7P
749.5359	Phosphatidylethanolamine	PEp 38:5	C43H76NO7P
749.5359	Phosphatidylethanolamine	PEp 38:5	$C_{43}H_{76}NO_7P$
751.5516	Phosphatidylethanolamine	PEp 38:4	C43H78NO7P
751.5516	Phosphatidylethanolamine	PEp 38:4	C43H78NO7P
753.5672	Phosphatidylethanolamine	PEp 38:3	C43H80NO7P
755.5829	Phosphatidylethanolamine	PEp 38:2	C43H82NO7P
757.5985	Phosphatidylethanolamine	PEp 38:1	C43H84NO7P
763.5152	Phosphatidylethanolamine	PE 38:6	C43H74NO8P
763.5152	Phosphatidylethanolamine	PE 38:6	C43H74NO8P
765.5309	Phosphatidylethanolamine	PE 38:5	$C_{45}H_{76}NO_8P$
765.5309	Phosphatidylethanolamine	PE 38:5	C43H76NO8P
767.5465	Phosphatidylethanolamine	PE 38:4	C43H78NO8P
773.5359	Phosphatidylethanolamine	PEp 40:7	C45H76NO7P
775.5516	Phosphatidylethanolamine	PEp 40:6	C45H78NO7P
777.5672	Phosphatidylethanolamine	PEp 40:5	$C_{45}H_{80}NO_7P$
779.5829	Phosphatidylethanolamine	PEp 40:4	C45H82NO7P
781.5985	Phosphatidylethanolamine	PEp 40:3	$C_{45}H_{84}NO_7P$
783.6142	Phosphatidylethanolamine	PEp 40:2	$C_{45}H_{86}NO_7P$
785.6298	Phosphatidylethanolamine	PEp 40:1	C45H88NO7P

789.5309	Phosphatidylethanolamine	PE 40:7	C45H76NO8P
791.5465	Phosphatidylethanolamine	PE 40:6	$C_{45}H_{78}NO_8P$
791.5465	Phosphatidylethanolamine	PE 40:6	C45H78NO8P
793.5622	Phosphatidylethanolamine	PE 40:5	$C_{45}H_{80}NO_8P$
795.5778	Phosphatidylethanolamine	PE 40:4	$C_{45}H_{84}NO_8P$
797.5935	Phosphatidylethanolamine	PE 40:3	$C_{45}H_{84}NO_8P$
799.6091	Phosphatidylethanolamine	PE 40:2	$C_{45}H_{86}NO_8P$
465.2855	Lysophosphatidylethanolamine	LPE 17:1	C22H44NO7P
425.2542	Lysophosphatidylethanolamine	LPE 14:0	C19H40NO7P
451.2699	Lysophosphatidylethanolamine	LPE 16:1	C21H42NO7P
453.2855	Lysophosphatidylethanolamine	LPE 16:0	C21H44NO7P
479.3012	Lysophosphatidylethanolamine	LPE 18:1	C23H46NO7P
481.3168	Lysophosphatidylethanolamine	LPE 18:0	C23H48NO7P
501.2855	Lysophosphatidylethanolamine	LPE 20:4	C25H44NO7P
503.3012	Lysophosphatidylethanolamine	LPE 20:3	C25H46NO7P
505.3168	Lysophosphatidylethanolamine	LPE 20:2	C25H48NO7P
507.3325	Lysophosphatidylethanolamine	LPE 20:1	C25H50NO7P
509.3481	Lysophosphatidylethanolamine	LPE 20:0	C25H52NO7P
499.2699	Lysophosphatidylethanolamine	LPE 20:5	C25H42NO7P
525.2855	Lysophosphatidylethanolamine	LPE 22:6	C27H44NO7P
527.3012	Lysophosphatidylethanolamine	LPE 22:5	$C_{27}H_{46}NO_7P$
529.3168	Lysophosphatidylethanolamine	LPE 22:4	C27H48NO7P
666.4472	Phosphatidylglycerol	PG 28:0	C34H67O10P
720.4941	Phosphatidylglycerol	PG 32:1	C38H73O10P
722.5098	Phosphatidylglycerol	PG 32:0	C38H75O10P
746.5098	Phosphatidylglycerol	PG 34:2	C40H75O10P
748.5254	Phosphatidylglycerol	PG 34:1	C40H77O10P
750.5411	Phosphatidylglycerol	PG 34:0	C40H79O10P

770.5098	Phosphatidylglycerol	PG 36:4	C42H75O10P
772.5254	Phosphatidylglycerol	PG 36:3	C42H77O10P
774.5411	Phosphatidylglycerol	PG 36:2	$C_{42}H_{79}O_{10}P$
776.5567	Phosphatidylglycerol	PG 36:1	C42H81O10P
778.5724	Phosphatidylglycerol	PG 36:0	C42H83O10P
794.5098	Phosphatidylglycerol	PG 38:6	C44H75O10P
796.5254	Phosphatidylglycerol	PG 38:5	C44H77O10P
798.5411	Phosphatidylglycerol	PG 38:4	C44H79O10P
800.5567	Phosphatidylglycerol	PG 38:3	C44H81O10P
820.5254	Phosphatidylglycerol	PG 40:7	C46H77O10P
822.5411	Phosphatidylglycerol	PG 40:6	C46H79O10P
824.5567	Phosphatidylglycerol	PG 40:5	$C_{46}H_{81}O_{10}P$
826.5724	Phosphatidylglycerol	PG 40:4	C46H83O10P
810.5258	Phosphatidylinositol	PI 32:0	C41H79O13P
808.5102	Phosphatidylinositol	PI 32:1	C41H77O13P
806.4945	Phosphatidylinositol	PI 32:2	C41H75O13P
838.5571	Phosphatidylinositol	PI 34:0	C43H83O13P
836.5415	Phosphatidylinositol	PI 34:1	C43H81O13P
834.5258	Phosphatidylinositol	PI 34:2	C43H79O13P
866.5884	Phosphatidylinositol	PI 36:0	C45H87O13P
864.5728	Phosphatidylinositol	PI 36:1	C45H85O13P
862.5571	Phosphatidylinositol	PI 36:2	C45H83O13P
860.5415	Phosphatidylinositol	PI 36:3	C45H81O13P
858.5258	Phosphatidylinositol	PI 36:4	C45H79O13P
856.5102	Phosphatidylinositol	PI 36:5	C45H77O13P
890.5884	Phosphatidylinositol	PI 38:2	C47H87O13P
888.5728	Phosphatidylinositol	PI 38:3	C47H85O13P
886.5571	Phosphatidylinositol	PI 38:4	C47H83O13P

884.5415	Phosphatidylinositol	PI 38:5	C47H81O13P
882.5258	Phosphatidylinositol	PI 38:6	C47H79O13P
914.5884	Phosphatidylinositol	PI 40:4	C49H87O13P
912.5728	Phosphatidylinositol	PI 40:5	$C_{49}H_{85}O_{13}P$
910.5571	Phosphatidylinositol	PI 40:6	C49H83O13P
908.5415	Phosphatidylinositol	PI 40:7	C49H81O13P
679.4424	Phosphatidylserine	PS 28:0	C34H66NO10P
759.5050	Phosphatidylserine	PS 34:2	C40H74NO10P
761.5207	Phosphatidylserine	PS 34:1	C40H76NO10P
763.5363	Phosphatidylserine	PS 34:0	C40H78NO10P
781.4894	Phosphatidylserine	PS 36:5	C42H72NO10P
783.5050	Phosphatidylserine	PS 36:4	C42H74NO10P
785.5207	Phosphatidylserine	PS 36:3	C42H76NO10P
787.5363	Phosphatidylserine	PS 36:2	C42H78NO10P
789.5520	Phosphatidylserine	PS 36:1	C42H80NO10P
807.5050	Phosphatidylserine	PS 38:6	C44H74NO10P
809.5207	Phosphatidylserine	PS 38:5	C44H76NO10P
811.5363	Phosphatidylserine	PS 38:4	C44H78NO10P
813.5520	Phosphatidylserine	PS 38:3	C44H80NO10P
815.5676	Phosphatidylserine	PS 38:2	C44H82NO10P
817.5833	Phosphatidylserine	PS 38:1	C44H84NO10P
833.5207	Phosphatidylserine	PS 40:7	C46H76NO10P
835.5363	Phosphatidylserine	PS 40:6	C46H78NO10P
837.5520	Phosphatidylserine	PS 40:5	C46H80NO10P
839.5676	Phosphatidylserine	PS 40:4	C46H82NO10P
841.5833	Phosphatidylserine	PS 40:3	C46H84NO10P
843.5989	Phosphatidylserine	PS 40:2	C46H86NO10P
723.4591	Sulfatide	C12 Sulfatide	$C_{36}H_{69}NO_{11}S$

779.5217	Sulfatide	C16 Sulfatide	C40H77NO11S
795.5167	Sulfatide	C16-OH Sulfatide	C40H77NO12S
807.5530	Sulfatide	C18 Sulfatide	C42H81NO11S
823.5480	Sulfatide	C18-OH Sulfatide	C42H81NO12S
835.5843	Sulfatide	C20 Sulfatide	C44H85NO11S
851.5793	Sulfatide	C20-OH Sulfatide	C44H85NO12S
863.6156	Sulfatide	C22 Sulfatide	C46H89NO11S
879.6106	Sulfatide	C22-OH Sulfatide	C46H89NO12S
889.6313	Sulfatide	C24:1 Sulfatide	C48H91NO11S
905.6262	Sulfatide	C24:1-OH Sulfatide	C48H91NO12S
891.6469	Sulfatide	C24 Sulfatide	C48H93NO11S
907.6419	Sulfatide	C24-OH Sulfatide	C48H93NO12S
677.4996	Phosphatidylcholine	PC 28:0	C36H72NO8P
731.5465	Phosphatidylcholine	PC 32:1	C40H78NO8P
733.5622	Phosphatidylcholine	PC 32:0	C40H80NO8P
755.5465	Phosphatidylcholine	PC 34:3	C42H78NO8P
757.5622	Phosphatidylcholine	PC 34:2	C42H80NO8P
759.5778	Phosphatidylcholine	PC 34:1	C42H82NO8P
761.5935	Phosphatidylcholine	PC 34:0	C42H84NO8P
779.5465	Phosphatidylcholine	PC 36:5	C44H78NO8P
779.5465	Phosphatidylcholine	PC 36:5	C44H78NO8P
781.5622	Phosphatidylcholine	PC 36:4	C44H80NO8P
783.5778	Phosphatidylcholine	PC 36:3	C44H82NO8P
785.5935	Phosphatidylcholine	PC 36:2	C44H84NO8P
787.6091	Phosphatidylcholine	PC 36:1	C44H86NO8P
789.6248	Phosphatidylcholine	PC 36:0	C44H88NO8P
803.5465	Phosphatidylcholine	PC 38:7	C46H78NO8P
805.5622	Phosphatidylcholine	PC 38:6	C46H80NO8P

807.5778	Phosphatidylcholine	PC 38:5	C46H82NO8P
807.5778	Phosphatidylcholine	PC 38:5	C46H82NO8P
807.5778	Phosphatidylcholine	PC 38:5	C46H82NO8P
809.5935	Phosphatidylcholine	PC 38:4	C46H84NO8P
829.5622	Phosphatidylcholine	PC 40:8	C48H80NO8P
831.5778	Phosphatidylcholine	PC 40:7	C48H82NO8P
833.5935	Phosphatidylcholine	PC 40:6	C48H84NO8P
835.6091	Phosphatidylcholine	PC 40:5	C48H86NO8P
837.6248	Phosphatidylcholine	PC 40:4	C48H88NO8P
507.3325	Lysophosphatidylcholine	LPC 17:1	C25H50NO7P
509.3481	Lysophosphatidylcholine	LPC 17:0	C25H52NO7P
467.3012	Lysophosphatidylcholine	LPC 14:0	C22H46NO7P
493.3168	Lysophosphatidylcholine	LPC 16:1	C24H48NO7P
495.3325	Lysophosphatidylcholine	LPC 16:0	C24H50NO7P
519.3325	Lysophosphatidylcholine	LPC 18:2	C26H50NO7P
521.3481	Lysophosphatidylcholine	LPC 18:1	C26H52NO7P
523.3638	Lysophosphatidylcholine	LPC 18:0	C26H54NO7P
541.3168	Lysophosphatidylcholine	LPC 20:5	C28H48NO7P
543.3325	Lysophosphatidylcholine	LPC 20:4	C28H50NO7P
545.3481	Lysophosphatidylcholine	LPC 20:3	C28H52NO7P
547.3638	Lysophosphatidylcholine	LPC 20:2	C28H54NO7P
549.3794	Lysophosphatidylcholine	LPC 20:1	C28H56NO7P
551.3951	Lysophosphatidylcholine	LPC 20:0	C28H58NO7P
567.3325	Lysophosphatidylcholine	LPC 22:6	C30H50NO7P
646.5050	Sphingomyelin	C12 Sphingomyelin	C35H71N2O6P
702.5676	Sphingomyelin	C16 Sphingomyelin	C39H79N2O6P
716.5832	Sphingomyelin	C17 Sphingomyelin	C40H81N2O6P
730.5989	Sphingomyelin	C18 Sphingomyelin	C41H83N2O6P

758.6302	Sphingomyelin	C20 Sphingomyelin	C43H87N2O6P
784.6458	Sphingomyelin	C22:1 Sphingomyelin	$C_{45}H_{89}N_2O_6P$
786.6615	Sphingomyelin	C22 Sphingomyelin	C45H91N2O6P
812.6771	Sphingomyelin	C24:1 Sphingomyelin	C47H93N2O6P
814.6928	Sphingomyelin	C24 Sphingomyelin	C47H95N2O6P
842.7241	Sphingomyelin	C26 Sphingomyelin	C49H99N2O6P
840.7084	Sphingomyelin	C26:1 Sphingomyelin	C49H97N2O6P
704.5832	Sphingomyelin	C16DH Sphingomyelin	C39H81N2O6P
732.6145	Sphingomyelin	C18DH Sphingomyelin	C41H85N2O6P
760.6458	Sphingomyelin	C20DH Sphingomyelin	C43H89N2O6P
800.6407	Sphingomyelin	C22:1DH Sphingomyelin	$C_{45}H_{89}N_2O_7P$
788.6771	Sphingomyelin	C22DH Sphingomyelin	C45H93N2O6P
814.6928	Sphingomyelin	C24:1DH Sphingomyelin	C47H95N2O6P
816.7084	Sphingomyelin	C24DH Sphingomyelin	C47H97N2O6P
842.7241	Sphingomyelin	C26:1DH Sphingomyelin	C49H99N2O6P
844.7397	Sphingomyelin	C26DH Sphingomyelin	C49H101N2O6P
481.4495	Ceramide	C12 Cer; N-(dodecanoyl)-ceramide; N-(dodecanoyl)-ceramide	C30H59NO3
509.4808	Ceramide	C14 Cer; N-(tetradecanoyl)-ceramide; N-(myristoyl)-ceramide	C32H63NO3
537.5121	Ceramide	C16 Cer; N-(hexadecanoyl)-ceramide; N-(palmitoyl)-ceramide; N-palmitoyl-sphingosine	C34H67NO3
549.5121	Ceramide	C17 Cer; N-(heptadecanoyl)-ceramide	C35H67NO3
565.5434	Ceramide	C18 Cer; N-(octadecanoyl)-ceramide; N-(stearoyl)-ceramide	C36H71NO3
593.5747	Ceramide	C20 Cer; N-(eicosanoyl)-ceramide; N-(eicosanoyl)-ceramide	C38H75NO3
621.6060	Ceramide	C22 Cer; N-(docosanoyl)-ceramide; N-(docosanoyl)-ceramide; N-behenoyl-sphingosine	C40H79NO3
647.6216	Ceramide	C24:1 Cer; N-(15Z-tetracosenoyl)-ceramide; N-(15Z-tetracosenoyl)-ceramide; N-nervonyl-sphingosine	C42H81NO3
649.6373	Ceramide	C24 Cer; N-(tetracosanoyl)-ceramide; N-(tetracosanoyl)-ceramide; N-lignoceroyl-sphingosine	C42H83NO3
675.6529	Ceramide	C26:1 Cer; N-(17Z-hexacosenoyl)-ceramide; N-(17Z-hexacosenoyl)-ceramide	C44H85NO3
677.6686	Ceramide	C26 Cer; N-(hexacosanoyl)-ceramide; N-(hexacosanoyl)-ceramide	C44H87NO3

539.5277	Ceramide	C16DH Cer; N-(hexadecanoyl)-dihydroceramide; N-(palmitoyl)-dihydroceramide	C34H69NO3
567.5590	Ceramide	C18DH Cer; N-(octadecanoyl)-dihydroceramide; N-(stearoyl)-dihydroceramide	C36H73NO3
595.5903	Ceramide	C20DH Cer; N-(eicosanoyl)-dihydroceramide; N-(eicosanoyl)-dihydroceramide	C38H77NO3
623.6216	Ceramide	C22DH Cer; N-(docosanoyl)-dihydroceramide; N-(docosanoyl)-dihydroceramide	C40H81NO3
651.6529	Ceramide	C24DH Cer; N-(tetracosanoyl)-dihydroceramide; N-(tetracosanoyl)-dihydroceramide	C42H85NO3
649.6373	Ceramide	C24:1DH Cer; N-(15Z-tetracosenoyl)-dihydroceramide; N-(15Z-tetracosenoyl)-dihydroceramide; N-nervonyl-sphinganine	C42H83NO3
679.6842	Ceramide	C26DH Cer; N-(hexacosanoyl)-dihydroceramide; N-(hexacosanoyl)-dihydroceramide	C44H89NO3
677.6686	Ceramide	C26:1DH Cer; N-(17Z-hexacosenoyl)-dihydroceramide; N-(17Z-hexacosenoyl)-dihydroceramide	C44H87NO3
638.6001	Cholesteryl Ester	17:0 Cholesteryl ester	$C_{44}H_{78}O_2$
666.6315	Cholesteryl Ester	19:0 Cholesteryl ester	C46H82O2
624.5845	Cholesteryl Ester	16:0 Cholesteryl ester	C43H76O2
652.6158	Cholesteryl Ester	18:0 Cholesteryl ester	C45H80O2
650.6002	Cholesteryl Ester	18:1 Cholesteryl ester	C45H78O2
648.5845	Cholesteryl Ester	18:2 Cholesteryl ester	C45H76O2
646.5689	Cholesteryl Ester	18:3 Cholesteryl ester	C45H74O2
674.6002	Cholesteryl Ester	20:3 Cholesteryl ester	C47H78O2
672.5845	Cholesteryl Ester	20:4 Cholesteryl ester	C47H76O2
670.5689	Cholesteryl Ester	20:5 Cholesteryl ester	C47H74O2
700.6158	Cholesteryl Ester	22:4 Cholesteryl ester	C49H80O2
698.6002	Cholesteryl Ester	22:5 Cholesteryl ester	C49H78O2
698.6002	Cholesteryl Ester	22:5 Cholesteryl ester	$C_{49}H_{78}O_2$
696.5845	Cholesteryl Ester	22:6 Cholesteryl ester	C49H76O2
386.3549	Cholesterol	Cholesterol	C27H46O
806.7363	Triacylglycerol	TG 48:0	C51H98O6
802.7050	Triacylglycerol	TG (48:2)	C51H94O6
804.7207	Triacylglycerol	TG (48:1)	C51H96O6

834.7676	Triacylglycerol	TG (50:0)	C53H102O6
832.7520	Triacylglycerol	TG (50:1)	C53H100O6
830.7363	Triacylglycerol	TG (50:2)	C53H98O6
828.7207	Triacylglycerol	TG (50:3)	C53H96O6
826.7050	Triacylglycerol	TG (50:4)	C53H94O6
860.7832	Triacylglycerol	TG (52:1)	C55H104O6
858.7676	Triacylglycerol	TG (52:2)	C55H102O6
856.7520	Triacylglycerol	TG (52:3)	C55H100O6
854.7363	Triacylglycerol	TG (52:4)	C55H98O6
852.7207	Triacylglycerol	TG (52:5)	C55H96O6
886.7989	Triacylglycerol	TG (54:2)	C57H106O6
884.7832	Triacylglycerol	TG 54:3)	C57H104O6
882.7676	Triacylglycerol	TG (54:4)	C57H102O6
880.7520	Triacylglycerol	TG (54:5)	C57H100O6
878.7363	Triacylglycerol	TG 54:6)	C57H98O6
906.7676	Triacylglycerol	TG (56:6)	C59H102O6
908.7832	Triacylglycerol	TG (56:5)	C59H104O6
910.7989	Triacylglycerol	TG (56:4)	C59H106O6
912.8145	Triacylglycerol	TG (56:3)	C59H108O6

Supplementary Figure

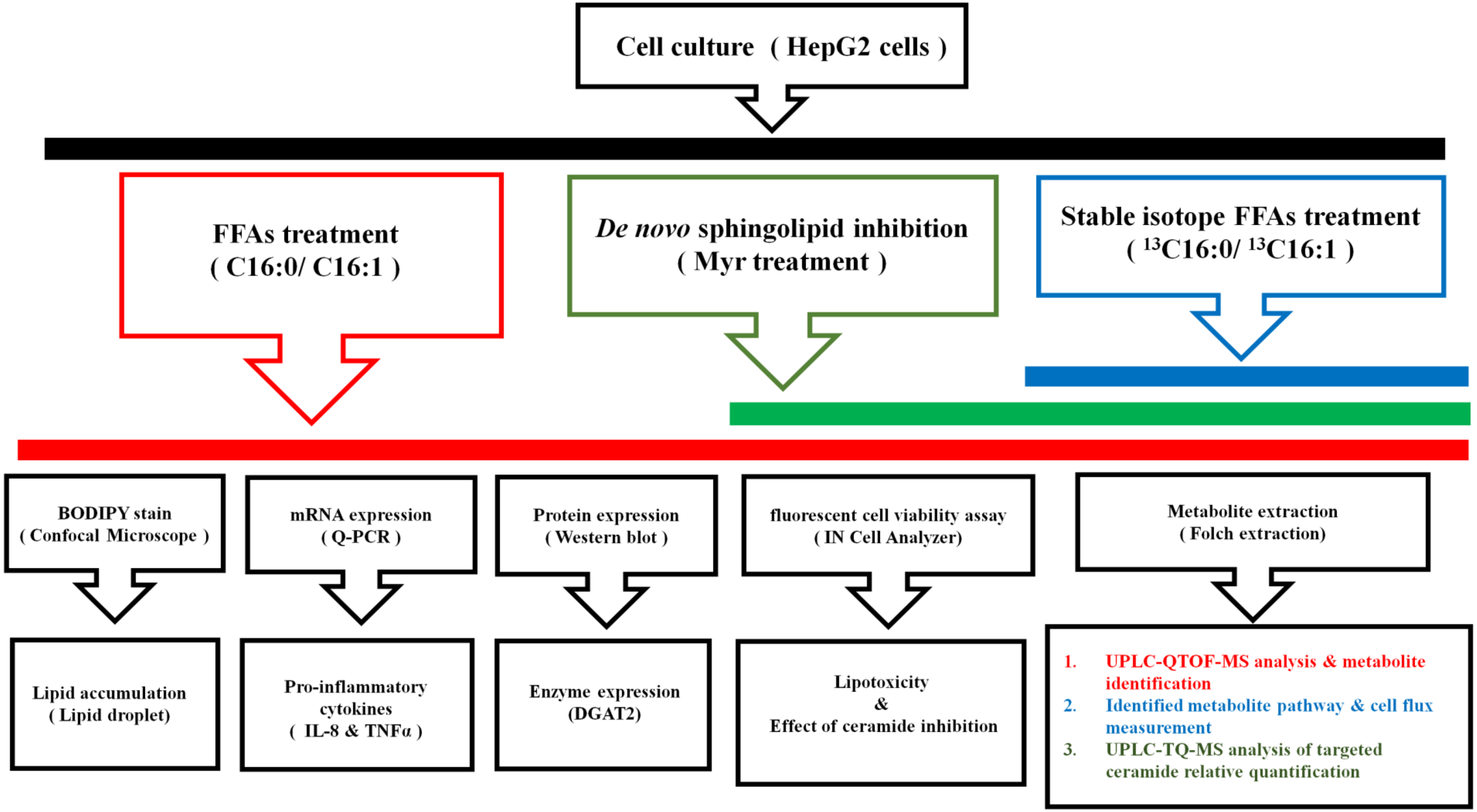

Figure S1. The workflow of FFAs treated HepG2 cell experiments

Supplementary materials and methods

Sample preparation for aqueous phase

In brief, aqueous phase was extracted from FFAs treated 10^6 HepG2 cells using the modified Folch method. The aqueous phase extract (upper layer) was dissolved in isopropanol/acetonitrile/water (2:1:1, V/V/V) mixture. After vortexing (30 sec, 4 times) and centrifuging (12,000 rpm, for 20 min at 4°C) the mixture, the supernatant was transferred into a vial for LC–MS analysis. [Samples per group were biological triplicates (n= total number of biological replicates) and each of biological triplicates was detected three times for technical triplicates (triplicates).]

Aqueous phase analyzed by LC-MS

Mass spectrometry analysis was performed using the ultra-performance liquid chromatography (UPLC) system coupled with time-of-flight mass spectrometry (TOF-MS; Waters, USA). Chromatographic separation was performed on an ACQUITY UPLC BEH Amide column (2.1 mm × 150 mm × 1.7 µm). Column temperature was maintained at 45°C. For metabolite profiling, the mobile phase A was water with 0.1% formic acid and the mobile phase B was acetonitrile with 0.1% formic acid. The flow rate was 0.4 mL/min, and the solvent gradient was as follows: 0–0.1 min, 99% solvent B; 0.1–7 min, 99%–30% solvent B; 7–7.2 min, 30%–99% solvent B; 7.2–10 min.

Mass spectrometric analysis was performed using the Waters Synapt HDMS system operating in positive- and negative-ion ESI mode. The capillary voltage was set at 2,700 V in ESI-positive mode and 2,000 V in ESI-negative mode and cone voltage was set at 35 V, respectively. Desolvation gas flow rate was set at 700 L/hr, and cone gas flow was maintained at 25 L/hr. The desolvation and source temperatures were set at 80°C. MS data were collected in centroid mode over a range of 20–990 m/z at a rate of 0.1 scan/sec. Leucine-enkephalin was used as the reference compound. LockSpray frequency was set at 0.5 sec and was averaged over 10 scans for correction.

Data matrices were determined utilizing the Progenesis QI software (Waters) by the extracted m/z value, retention time (RT), and ion intensity. Metabolite identification was performed by searching the extracted data against the METLIN (http://metlin.scripps.edu/index.php), LIPID MAPS (http://www.lipidmaps.org/), Human Metabolome Database (HMDB) (http://www.hmdb.ca/), and in-house database (Table S6).

© 2018 by the authors. Submitted for possible open access publication under the terms and conditions of the Creative Commons Attribution (CC BY) license (http://creativecommons.org/licenses/by/4.0/).
